# Metabolic Links to Socioeconomic Stresses Uniquely Affecting Ancestry in Normal Breast Tissue at Risk for Breast Cancer

**DOI:** 10.3389/fonc.2022.876651

**Published:** 2022-06-27

**Authors:** Denys Rujchanarong, Danielle Scott, Yeonhee Park, Sean Brown, Anand S. Mehta, Richard Drake, George E. Sandusky, Harikrishna Nakshatri, Peggi M. Angel

**Affiliations:** ^1^ Department of Cell and Molecular Pharmacology & Experimental Therapeutics, Bruker-MUSC Center of Excellence, Clinical Glycomics, Medical University of South Carolina, Charleston, SC, United States; ^2^ Department of Biostatistics and Medical Informatics, University of Wisconsin-Madison, Madison, WI, United States; ^3^ Department of Surgery, Indiana University School of Medicine, Indianapolis, IN, United States; ^4^ Department of Biochemistry and Molecular Biology, Indiana University School of Medicine, Indianapolis, IN, United States; ^5^ Department of Pathology and Laboratory Medicine, Indiana University School of Medicine, Indianapolis, IN, United States

**Keywords:** breast cancer risk, N-glycosylation, body mass index (BMI), glycomics mass spectrometry, ancestry, breast cancer disparities

## Abstract

A primary difference between black women (BW) and white women (WW) diagnosed with breast cancer is aggressiveness of the tumor. Black women have higher mortalities with similar incidence of breast cancer compared to other race/ethnicities, and they are diagnosed at a younger age with more advanced tumors with double the rate of lethal, triple negative breast cancers. One hypothesis is that chronic social and economic stressors result in ancestry-dependent molecular responses that create a tumor permissive tissue microenvironment in normal breast tissue. Altered regulation of N-glycosylation of proteins, a glucose metabolism-linked post-translational modification attached to an asparagine (N) residue, has been associated with two strong independent risk factors for breast cancer: increased breast density and body mass index (BMI). Interestingly, high body mass index (BMI) levels have been reported to associate with increases of cancer-associated N-glycan signatures. In this study, we used matrix assisted laser desorption/ionization (MALDI) imaging mass spectrometry (IMS) to investigate molecular pattern changes of N-glycosylation in ancestry defined normal breast tissue from BW and WW with significant 5-year risk of breast cancer by Gail score. N-glycosylation was tested against social stressors including marital status, single, education, economic status (income), personal reproductive history, the risk factors BMI and age. Normal breast tissue microarrays from the Susan G. Komen tissue bank (BW=43; WW= 43) were used to evaluate glycosylation against socioeconomic stress and risk factors. One specific N-glycan (2158 m/z) appeared dependent on ancestry with high sensitivity and specificity (AUC 0.77, Brown/Wilson p-value<0.0001). Application of a linear regression model with ancestry as group variable and socioeconomic covariates as predictors identified a specific N-glycan signature associated with different socioeconomic stresses. For WW, household income was strongly associated to certain N-glycans, while for BW, marital status (married and single) was strongly associated with the same N-glycan signature. Current work focuses on understanding if combined N-glycan biosignatures can further help understand normal breast tissue at risk. This study lays the foundation for understanding the complexities linking socioeconomic stresses and molecular factors to their role in ancestry dependent breast cancer risk.

## Introduction

A primary difference between Black women (BW) and White women (WW) diagnosed with breast cancer is the aggressiveness of the tumor (1–5). Not only do BW have higher mortalities with similar incidence of breast cancer compared to other genetic ancestries, but they are also diagnosed at younger ages with more advanced tumors including double the rates of lethal, triple negative breast cancers (TNBC) (2, 4–8). Contributing factors to this discrepancy include, but are not limited to, disparities in income, barriers to screening opportunities, differences in the quality of treatment, higher stages of disease at diagnosis and elevated incidence of TNBC (1, 5, 6, 8–12). Additionally, these factors can also contribute to BW experiencing an unequal burden of co-morbid diseases (e.g. obesity, diabetes and hypertension) that correlate overall differences in lifestyles as well as barriers to medical services (4, 11–13).

A study assessing prioritization of breast cancer risk factors determined that body mass index (BMI) and weight gain was listed as second only to Gail Model parameters [quantitative breast density, free estradiol, parity (yes/no), and age of menopause] in importance (14–16). Unfortunately, BW have the highest rates of overweight and obese BMI categories relative to other genetic ancestries in the United States (4, 11, 13, 17); compared to non-Hispanic whites, BW are about 50% more likely to be obese (9, 17). This is problematic as obesity and/or elevated BMI are associated with poorer breast cancer prognosis and/or increased mortality in both premenopausal and postmenopausal women (14, 18). In obese postmenopausal women, adipose tissue can act as the main source of estrogen biosynthesis (19); the higher the amount of adipose tissue the higher the levels of estrogen, thus the higher the risk for breast cancer (14, 20). Additionally, the increase in adipose tissue can also lead to a pro-inflammatory microenvironment that is activated and sustained by the nuclear factor kappa B (NF-kB) pathway (14, 19, 21, 22). NF-kB is constitutively active in many cancers and aids in the secretion of leptin, pro-inflammatory cells and cytokines like interleukins-1β (IL-1β) and interleukin 6 (IL-6) (22). In endothelial cells, a pro-inflammatory microenvironment can help accelerate cell proliferation, inhibit apoptosis, promote cell migration and invasion, and induce changes in cell-surface protein N-glycosylation, a glucose metabolism-linked post-translational modification (22–26).

Glycoproteins are abundant on cell surfaces and serve as one of the initial points of contact in orchestrated interactions that mediate cell-cell, cell-matrix and cell-molecule cross-talk in normal and cancerous tissue (24, 25, 27, 28). N-glycans play important roles in cell mobility, cell growth, intracellular signaling, metastatic capacity, and cellular immune properties (27, 29–31). Previous studies have demonstrated the importance of studying glycosylation and its regulation in breast cancer progression, as systemic changes in glycosylation are nonrandom and represent a hallmark of cancer progression (24, 29–37). However, equally important is understanding their implications in breast cancer control including risk, early detection, prognosis and therapeutic targets.

Aberrant alteration of N-glycosylation plays a significant role throughout breast cancer progression and influences clinical outcome (33, 38–47). Over many cancer types, changes to N-glycan sugar residue composition and branching structure contributes to processes including stroma-cell adhesive interactions, migration, immune recruitment, and malignant conversion (27, 29, 31, 33, 48). Increases in branching, outer arm fucosylation, and sialylation have all been associated with clinical cases of breast cancer and link to breast cancer subtypes (40–42, 49). In breast cancer, modifications of cell surface 2,6-sialylation alters cell-cell and cell-extracellular matrix (ECM) adhesion (50); loss of adhesive interactions is one of the first steps in metastatic pathways (31, 51). In advanced breast cancer, N-glycan complexity increases; N-acetyllactosamines have been associated with advanced HER2+ and triple negative breast cancer (43, 45). Additionally, a separate study found a core-fucosylated tetra-antennary glycan containing a single N-acetyllactosamine was associated with poor clinical outcomes in breast cancer (38). Throughout progression to metastasis, multiple genes along the N-glycan synthesis pathways are predictive of breast cancer diagnosis and indicators of breast cancer outcomes (33, 41, 52, 53). Notably although decades of research have pointed to contributions of glycosylation in breast cancer, studies evaluating breast tissue from donors at risk for breast cancer are rare. A single study reported that increases in breast density by mammography were associated with increases in biantennary digalactosylated glycans and decreases in trisialylated or outer-arm fucosylated glycans (49). Interestingly, this same study reported that high BMI increases cancer-like N-glycan features, trisialylated, triantennary, and outer-arm fucosylated glycans (49), suggesting links between lifestyle and N-glycan expression patterns. However, very little remains known regarding the specific glycosylation alterations that are present in breast cancer risk.

While there is a clear and strong association between Black ancestry and poor breast cancer prognosis (1–3), the underlying molecular factors remain to be elucidated. There is ongoing debate regarding whether the underlying cause of higher mortality is related to healthcare inequalities or due to ancestry dependent molecular features found in normal breast tissue that facilitate differences in breast cancer outcomes (2, 12). In this study, we hypothesize that lifestyle and socioeconomic stresses influence metabolism to result in altered N-glycosylation associated with breast cancer risk. To test this, we investigate N-glycan profiles from normal breast tissue with significant 5-year risk of breast cancer based on Gail score. Comparisons are made based on genetic ancestry, body mass index (BMI), and socioeconomic factors including marital status, income and education status. A main finding is that obese BW have a specifically altered set of N-glycans that involves fucosylation. Initial tests to investigate socioeconomic factors link increases in specific N-glycans to different stressors based on generic ancestry. These studies increase our understanding of the molecular foundations of breast cancer risk towards halting cancer and decreasing the impact of cancer on the individual, family and community.

## Methods

### Materials

High‐performance liquid chromatography–grade acetonitrile, ethanol, methanol, xylene, and water were obtained from Fisher Scientific (Pittsburg, PA, USA). Citraconic anhydride for antigen retrieval was obtained from Thermo Scientific (Bellefonte, PA, USA). Alpha‐cyano‐4‐hydroxycinnamic acid (CHCA) and Trifluoroacetic acid (TFA) were obtained from Sigma‐Aldrich (St. Louis, MO, USA). Recombinant PNGaseF PRIME™ was obtained from N‐Zyme Scientifics (Doylestown, PA, USA). Hematoxylin and Eosin (H&E) stains were obtained from Cancer Diagnostics (Durham, NC, USA).

### Human Tissues

Archived normal breast tissue samples [BW n=43; WW n=43] were obtained from The Susan G. Komen for the Cure Tissue Bank at the IU Simon Cancer Center. A commercial breast cancer progression TMA was obtained from US Biomax, Inc. Use for the study was approved as exemption 4 status by the Medical University of South Carolina Institutional Review Board.

### Tissue Preparation

After H&E staining, tissues were de-stained and processed for N-glycan analysis (54–59). For de-staining, the tissues underwent a series of xylene, ethanol and Carnoy’s solution washes before the tissue was prepped for imaging. Antigen retrieval was then performed using citraconic anhydride buffer [25 µL citraconic anhydride (Thermo Fisher Scientific, Waltham, MA) 2 µL 12M HCl, 50 mL HPLC grade water, pH 3.0-3.5]. N-glycans were then released using PNGase PRIME™ (N-Zyme Scientifics, Doylestown, PA) applied using an HTX M3 TMSprayer (HTX Technologies LLC, Chapel Hill, NC) with the following parameters: 25 µL/min, 15 passes, 45°C, 1200 mm/min velocity, and 0 mm offset. Slides were incubated at 37°C in humidity chambers for 2 hours and briefly dried under vacuum. Once dry, 7mg/mL of CHCA (Sigma Aldrich, St. Louis, MO) matrix in 50% acetonitrile, 0.1% TFA was applied using the same TMSprayer set to 0.1 mL/min for 10 passes at 78°C with a velocity of 1300 mm/min and a 1 mm offset.

### N-Glycan Analysis Using MALDI-FT IMS

A Solarix dual source 7T FT-ICR mass spectrometer (Bruker Daltonik, Bremen, Germany) containing a SmartBeam II laser operating at 2000 Hz was used for analysis of released N-glycan ions. Ions were detected with a laser spot size of 25 µm using 200 laser shots per pixel with a stepsize of 100 µm. N-glycans were measured in positive ion mode with a 1.2059 s transient over mass range 700-5000. Data was analyzed using FlexImaging 5.0 and SCiLS Lab 2019c (Bruker Daltonik) normalized to total ion current. Exported peak intensities were transformed using natural log prior to statistical testing. Glycans annotated by accurate mass were assigned by hexose content. Putative structures are described using databases from previous imaging studies on human tissues using GlycoWorkbench (58, 60–67). Compositional accuracy of the glycan structures defined herein was determined based on accurate mass and prior structural characterizations by MALDI-FTICR MS, MALDI-TOF MS and LC-MS/MS (43, 45, 68).

### Statistical Analysis

GraphPad version 9.02 was utilized for receiver operating characteristics (ROC) curve analysis and Mann-Whitney U test analyses. A ROC curve is a plot of Sensitivity versus 1-Specificity which generates an area under the curve (AUC) used as an effective measure of accuracy (69). The Mann-Whitney U test is a nonparametric test used to compare medians between two populations (70). To investigate how the associations between N-Glycan and socioeconomic covariates (e.g., Gail score, age, BMI, Household income, College, Graduate, Single and Married indicators) differ across white and black groups, we applied a groupwise envelope model which is a multivariate linear regression model identifying the material part of the responses to the estimation of the regression coefficients and removing the immaterial part to make the estimation more efficient (71). Using R version 4.1.2, a Box’s M-test was conducted to test the homogeneity of covariance matrices between races. The covariance matrices between white and black groups are not significantly homogeneous (p-value= 9.752e-07). We fit a linear regression using a groupwise envelope model of the logarithm of N-glycans for 21 genes selected by Mass. All variables were standardized and the heatmaps of regression coefficients for each group were displayed to see the relationship. In all statistical analyses, *p*-values <0.05 were reported as significant.

## Results

### Patient Cohort Characteristics Show Black Women and White Women Differ in BMI and Gail Scores

The patient cohort was comprised of Black women (BW, n=43) and white women (WW, n=43) with high risk of breast cancer. Stroma rich normal breast tissue microarrays (TMAs) from BW and WW with mapped ancestry were used for glycomics mass spectrometry analysis (**Supplementary Figure 1, 2**; **Supplementary Table 1, 2**). One sample from the BW cohort and one sample from the WW cohort were removed in future analysis because of missing ancestry data. The two groups were compared based on age, body mass index (BMI), education level, Gail score, household income and marital status (**Tables 1**, **2**). A total of 13 BW samples and 13 WW samples did not have age, body mass index (BMI), education level, Gail score, household income and marital status information and were removed when assessing these factors between BW versus WW. Most of the factors being investigated were not significantly different between the two cohorts, with the exceptions of BMI [BW, n=30 Median BMI 32.2, 95% CI (29.63, 36.65); WW, n=30 Median BMI 24.45, 95% CI (24.45, 29.17); p-value = 0.002] and Gail scores [BW, n=30 Median Gail 9.40, 95% CI (8.43, 9.41); WW, n=30 Median Gail 10.35, 95% CI (9.91, 12.17); p-value<0.0001]. Further investigations and analysis in this study will be focused on BMI and Gail scores as important lifestyle factors to consider for racial disparities associated with breast cancer risk.

**Table 1:** Patient cohort characteristics. Median and confidence intervals [CIs] for age.

Characteristics	Black Women	White Woman	P- value
	(n=30)	(n=30)	
Age (Years)	42.5 [41.57 - 46.70]	42.0 [41.63 47.91]	0.92
Body Mass Index (BMI)	32.20 [29.63 -36.65]	24.45 [24.45-29.17]	0.002
Education Level	5.0 [4.55 -5.92]	5.0 [4.63-5.57]	0.582
Gail Score	9.40 [8.43 -9.41]	10.35 [9.91- 12.17]	<0.001
Household Income	3.0 [2.69 -3.31]	3.0 [2.89- 3.30]	0.764
Marital Status	2.0 [1.71-2.29]	2.0 [1.87-2.26]	0.489

Body Mass Index (BMI), Education level, Gail score. Household Income and Marital Status for Black women (n=30) and white woman (n=30). Education level scoring; less than high School (1); High school graduate or GED (2); Vocation or Technical school (3); Associate’s Degree (4); Bachelor’s Degree (5); Graduate Degree (6); Professional School (7); or Other (9). Household income scoring; < $20,000 (1); 20,001 – 50,000 (2); $50,001 – 100,000 (3); >$100, 000; Prefer not to answer (9). Marital status scoring: Single (1); Married (2); Divorced (3); Widowed (4).

**Table 2:** Education level, household income, and marital status scoring categories.

Education Level	
Less than High School	1
high Scholl graduate or GED	2
Vocation or techinical school	3
Associate's Degree	4
Bachelor's Degree	5
Graduate Degree (Masters or Doctorate)	6
Professional School (MD or Lawyer)	7
Other (some college, certificate program, apprenticeship)	9
**Household Income**	
Under 20, 000	1
20,.001- to 50,000	2
50,001 to 100,000	3
More than 100,000	4
Prefer not to answer	9
**Marital Status**	
Single	1
Married	2
Divorced	3
Widowed	4

### N-Glycan Imaging of Normal Breast TMAs Reveal Distinct N-Glycan Peak Intensity Patterns

**Figure 1:**
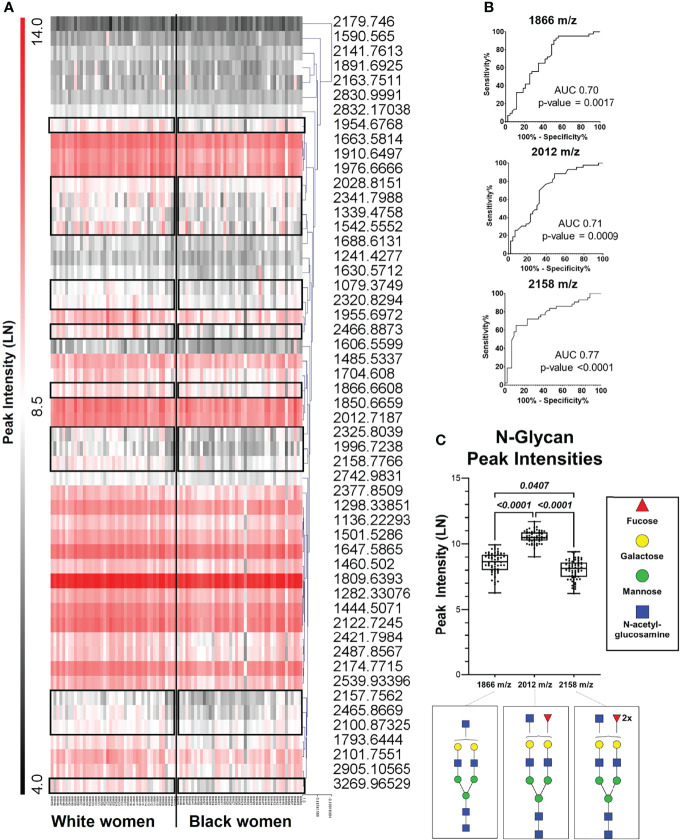
Specific N- Glycan patterns are observed among women at risk for breast cancer. **(A)** Hierarchal cluster heat map of 53 N-glycan from all patient samples with black boxes highlighting differences between Black women and white women. **(B)** Receiver operating curve (ROC) analysis of peaks 1866 m/z, 2012 m/z, and 2158 m/z showed area under the curve (AUC) values of 0.70, 0.71 and 0.77, respectively (p- value <0.005). **(C)** Peak intensity box plots of peaks 1866 m/z (HexHexNAc5), 2012 m/z (Hex5dHex1HexNAc5), and 2158 m/z (Hex5dHexHexNAc5)corresponding to a non-fucosylated, singly- fucosylated biantennary N-glycans.

N-glycans are constantly regulated based on environmental and molecular factors (27). In order to understand the N-glycan alterations that may contribute to a breast cancer risk, the distribution of N-glycans were defined in normal breast tissues from BW and WW donors. A total of 53 N-glycans were identified using MALDI FT-ICR imaging mass spectrometry analysis on TMAs from BW and WW cohorts (**Figure 1**). A hierarchal cluster heat map of the total N-glycans demonstrates differential N-glycan peak intensity among all patients (**Figure 1A**). Receiver operating characteristics (ROC) curve analysis highlights 23 out of 53 N-glycans generated significant area under the curve (AUC) values when looking at BW versus WW (p-value <0.005) (**Supplementary Table 3**). ROC curve analysis between BW and WW revealed that out of all the glycans with a determined composition N-glycan 2158.777 m/z, Hex5dHex2HexNAc5 (2158 m/z) had the highest AUC (AUC 0.77; p-value <0.0001). Further analysis will focus on doubly-fucosylated N-glycan 2158 m/z and its variations that differ in the numbers of fucose (146 m/z) residues. In addition to 2158 m/z, ROC curve analysis between BW and WW had significant AUC values (p-value < 0.005) for N-glycans 1866.661 m/z, Hex5HexNAc5 (1866 m/z; AUC 0.70) and 2012.719 m/z, Hex5dHex1HexNAc5 (2012 m/z; AUC 0.71) (**Figure 1B**). Differential peak intensity patterns were observed in specific N-glycans 1866 m/z, 2012 m/z, and 2158 m/z that correspond to a non-fucosylated, singly-fucosylated, and doubly-fucosylated bianntenary N-glycans, respectively (**Figure 1C**). Overall, specific N-glycan intensity patterns are observed among women at risk for breast cancer.

### Unique N-Glycan Peak Intensity Patterns Are Associated With Specific Socioeconomic Stresses in an Ancestry Dependent Manner

**Figure 2:**
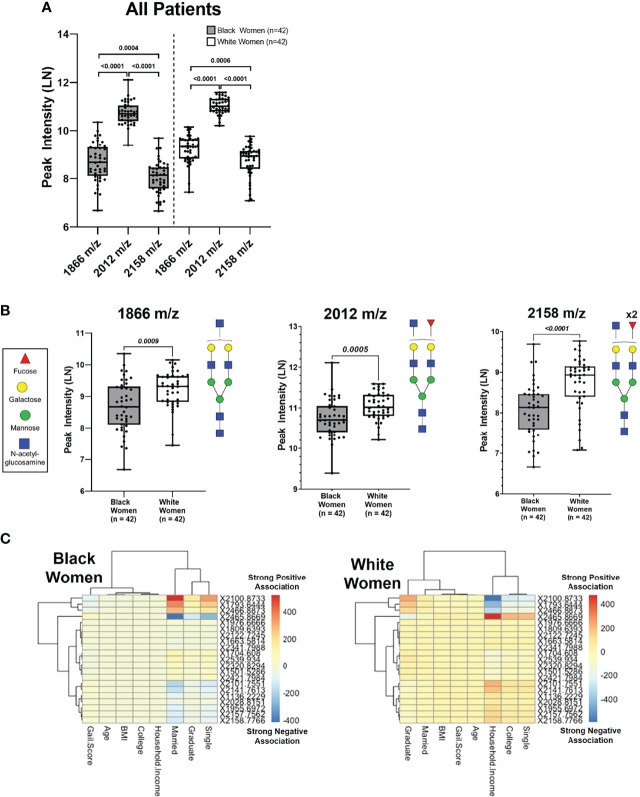
Unique N-Glycan peak intensity patterns are associated with Black women and white women. **(A)** Peak intensities of N-glycans 1866 m/z, 2012 m/z and 2158 m/z analyzed from Black and White women. Each data point represents a patient (Black n=42 and White n=42). **(B)** Comparison of N-glycan peak intensifies show white women have higher intensifies compared to Black women for peaks 1866 m/z (p= 0.0009), 2012 m/z (p=0.0005) and 2158 m/z (p < 0.001) **(C)** Linear regression model with ancestry as group variable & socioeconomic covariates as predictors (p <0.0001). The numbers 400 (Strong Negative Association) on the color bar indicate the values of the regression coefficients.

Ancestry has been shown to influence breast cancer risk (6, 72–77), however, less is known about ancestry-dependent changes in N-glycan regulation. To determine if differential N-glycan profile patterns observed in women are influenced by genetic ancestry, the same 53 N-glycans were further assessed between BW and WW. The mass spectra demonstrate slight differences in N-glycan peak intensities between BW and WW (**Supplementary Figure 3**). When looking at the three biantennary N-glycans, 2012 m/z demonstrated significantly higher intensity compared to 1866 m/z and 2158 m/z in both cohorts of women (p-value < 0.0001) (**Figure 2A**).

When comparing N-glycan peak intensities between BW and WW, 2012 m/z was significantly upregulated in WW versus BW (**Figure 2B**). This trend of upregulated N-glycan peak intensities in WW relative to BW was maintained among the non-fucosylated 1866 m/z and doubly fucosylated 2158 m/z N-glycan peaks (p-value < 0.001; **Figure 2B**). The data suggests that women with high risk of breast cancer have distinct biantennary N-glycan expression patterns with potential ancestry-dependent influences.

Fitting a linear regression called a groupwise envelope model of N-Glycan on socioeconomic covariates with ancestry as group variables, N-glycan patterns were examined in relation to social and economic stresses by ancestry (p-value = 9.752E-7). Certain N-glycans were strongly associated with household income for WW, while the same N-glycans were strongly associated with marital status for BW (**Figure 2C**). The data suggests that metabolic patterns linked to socioeconomic stresses may contribute to ancestry-dependent breast cancer risk.

### Differential N-Glycan Peak Intensities Are Observed Based on Ancestry and BMI

An important contributing factor of breast cancer risk that must be considered is a patient’s body mass index (BMI), as obesity affects specific genetic ancestry groups more than others (4, 11, 13, 17, 19, 78). While changes in glycosylation have been implicated in cancer progression, the relationship between BMI, glycosylation regulation and metabolomics has yet to be studied with respect to breast cancer risk. In order to further analyze potential contributing factors associated with the differential N-glycan profile intensities between BW and WW, the impact of BMI was explored.

**Figure 3:**
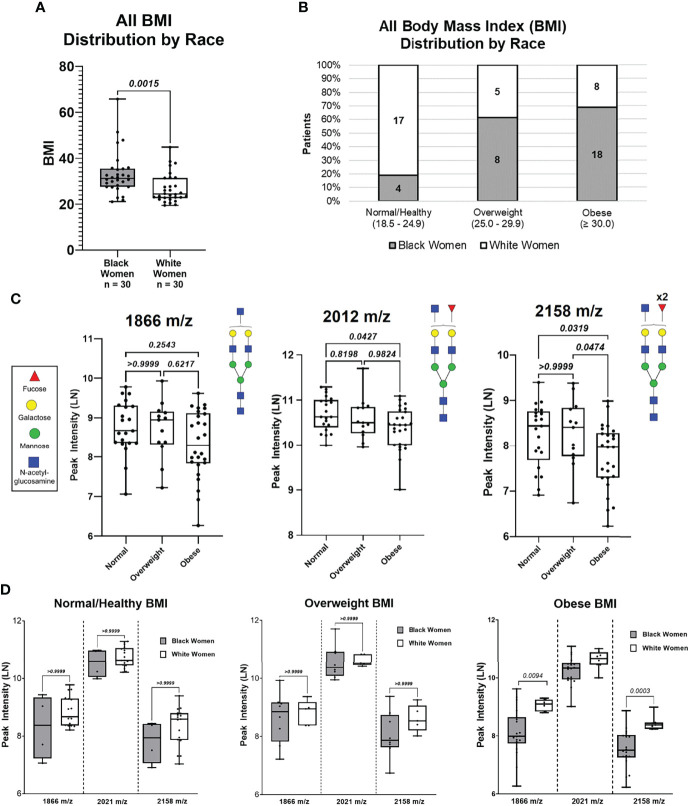
N-Glycan peak intensify analysis based on BMI (Normal, Overweight and Obese) of all patients. **(A)** Distribution of patient BMIs between Black women and White women cohorts. **(B)** Distribution of Black women and White patients based on BMI categories: Normal (18.5 – 24.9). Overweight (25.0 – 24.9), and Obese (≥30.0). **(C)** Comparing peak intensifies between BMI categories for N-glycans 1866 m/z and 2158 m/z based on BMI categories.

Based on our patient cohort, BW had an overall higher BMI average compared to WW (p-value = 0.002; **Figure 3A**). BW and WW were categorized based on BMI groups for clinical relevance (**Figure 3B**). The BMI categories included Normal/Healthy (BMI: 18.5 – 24.9; BW = 4, WW = 17), Overweight (BMI: 25.0 – 29.9; BW = 8, WW = 5), and Obese (BMI: ≥ 30; BW = 18, WW = 8). The same N-glycan peaks (1866 m/z, 2021 m/z, and 2158 m/z) were analyzed based on the BMI categories. Regardless of ancestry, peak intensity for 2012 m/z was significantly lower in the Obese compared to the Normal BMI group (**Figure 3C**). Additionally, the 2158 m/z N-glycan peak intensity was significantly lower in the Obese BMI group compared to both Normal and Overweight BMI groups. BMI categories were then compared between the BW and WW cohorts. N-glycan peaks 1866 m/z and 2158 m/z showed differential regulation only within the Obese group; Obese WW had significantly higher intensities of both N-glycans compared to the Obese BW (p-value <0.01; **Figure 3D**). These data suggest that the differential N-glycan patterns observed between the two cohorts may be influenced by ancestry and/or BMI.

### N-Glycan Intensity Peak Patterns Differ by Ancestry Even While Controlling for BMI, Gail Score and Menopausal Status

**Figure 4:**
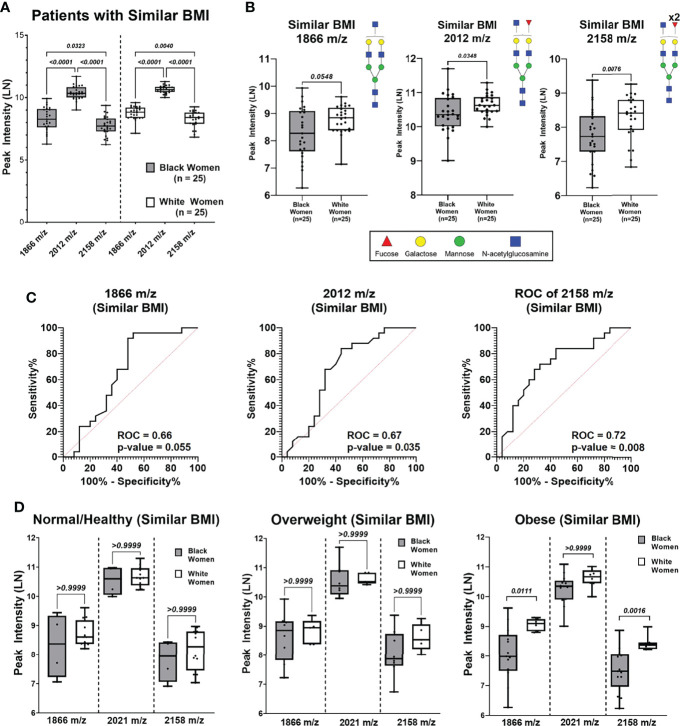
N-Glycan peak intensify analysis based on BMI (Normal, Overweight and Obese) of patient cohort with similar BMIs. **(A)** Comparing peak intensifies between N-glycans 1866 m/z, 2012 m/z from Black and White cohorts with similar BMIs. **(B)** Comparison of N-glycan peak intensifies show WW have higher intensifies compared to BW for peaks 1866 m/z (p = 0.0548), 2012 m/z (p = 0.0348) and 2158 m/z (p = 0.0076) **(C)** Receiver operating curve (ROC) analysis of peaks 1866 m/z, 2012m/z, and 2158m/z of patient cohort with similar BMIs show area under the curve (AUC) values of 0.66 (p-value = 0.055),0.67 (p-value = 0.035), and 0.72 (p-value = 0.008), respectively. **(D)** Comparing Black and White women peak intensifies of N-glycans 1866 m/z, 2012 m/z and 2158 m/z based on BMI categories.

To determine if the observed differential N-glycan peak intensities are BMI dependent, N-glycan profiles of BW (n=25) and WW (n=25) with similar BMI distributions were analyzed (**Supplementary Figure 4**; **Supplementary Table 4, 5**). Five BW from the Obese group and five WW from the Normal/Healthy group were removed to create a data subset of women with similar BMI distributions (p-value = 0.145; **Supplementary Figure 5A**). After controlling for BMI, BW and WW were again categorized based on BMI groups for clinical relevance (**Supplementary Figure 5B**). Patient specific peak intensities for 1866 m/z, 2012 m/z and 2158 m/z show consistent trends as the original data where the intensity for 2012 m/z is significantly higher compared to 1866 m/z and 2158 m/z (**Figure 4A**). Comparing the three N-glycan intensities by ancestry shows that WW have higher N-glycan intensities for 1866 m/z, 2012 m/z and 2158 m/z relative to BW N-glycan intensities (**Figure 4B**). ROC curve analysis reveals high AUC values for N-glycans 1866 m/z (AUC 0.66; p-value 0.055), 2012 m/z (AUC 0.67; p-value 0.035) and 2158 m/z (AUC 0.72; p-value 0.008) in the subgroup of patients with same BMI distributions (**Figure 4C**). Within the Obese group, N-glycans 2158 m/z (p-value 0.0016) and 1866 m/z (p-value 0.0111) remained significantly different between BW and WW even when controlling for BMI (**Figure 4D**). This data suggests that the significant differential peak expression observed between the Obese BW and WW may be ancestry-dependent.

To assess if the observed differential N-glycan intensities are dependent on Gail Score, BW (n=26) and WW (n=10) with similar Gail Scores (p-value = 0.840) were created as a subgroup (**Supplementary Figures 6, 7**; **Supplementary Tables 6, 7**). The three biantennary N-glycans did not show differential expression between the BW and WW when controlling for Gail Score (**Supplementary Figure 6C**).

Lastly, the menopausal status for each woman was included and showed that majority of the women were pre-menopausal (BW = 38, WW = 35) compared to post-menopausal (BW = 4, WW = 5). N-glycans 1866 m/z, 2012 m/z and 2158 m/z were then compared between BW and WW based on their menopausal status (**Supplementary Figure 8**). In the pre-menopausal group, WW had significantly higher relative peak intensities relative to BW for 1866 m/z (p = 0.0048), 2012 m/z (p = 0.0012) and 2158 m/z (p <0.0001) while no significant differences were observed in the post-menopausal group. On average, BW reach menopause about two years earlier (49 years) than the national median age (51 years) (79). It is also important to note that basal cell-like subtypes of breast cancer are more common in young pre-menopausal BW compared to post-menopausal BW and WW (80, 81).

### Similar N-Glycan Peak Glycan Trends in Breast Cancer Risk Are Detected in Breast Cancer Progression TMAs

**Figure 5:**
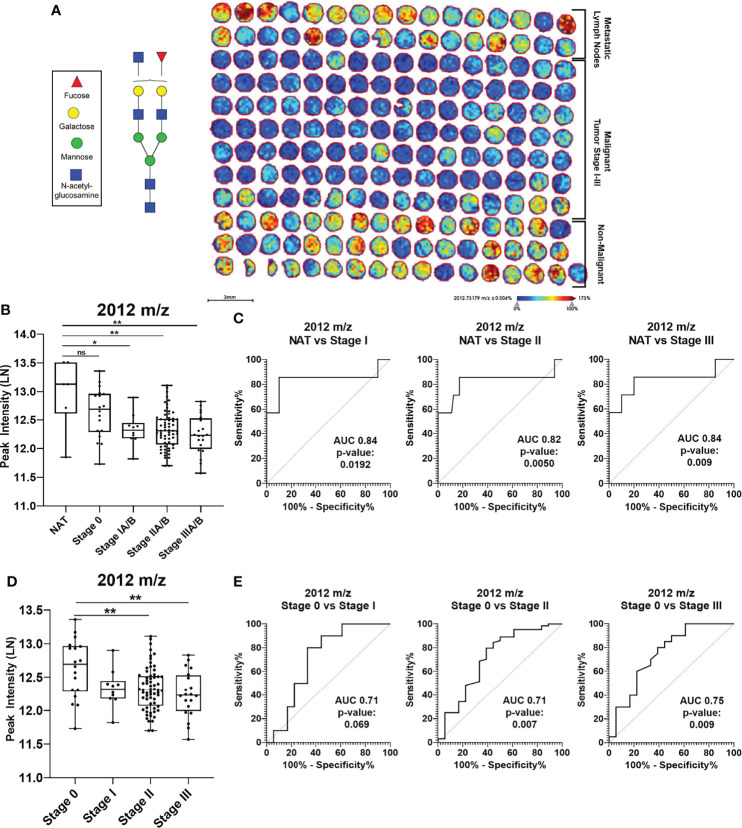
N-glycan 2012 m/z has specific intensify patterns in breast cancer progression. **(A)** Imaging peak intensify heat map of breast cancer progression TMAS for 2012 m/z in Benign, Inflammation, Hyperplasia, normal adjacent to the tumor (NAT), Adjacent to the tumor (AT), Stages 0-III, and Metastatic Lymp TMA cores. **(B)** Mann Whitney analysis of N-glycan peak 2012 m/z comparing intensifies between Normal Adjacent to the Tumor (NAT) and malignant cores Stages 0-III (* = p-value<0.05; ** =p-value <0.01). **(C)** ROC curve analysis for peak. 2012 m/z looking at NAT vs Stage IIIA/B (ROC AUC 0.847, p-value 0.009). **(D)** Mann Whitney analysis of N-glycan peak 2012 m/z comparing intensifies between Stages 0-III (** = p- value <0.01). **(E)** ROC curve analysis for peak 2012 m/z looking at Stage 0 vs stage IA/B (ROC AUC 0.71, p- value 0.069), Stage 0 vs Stage IIA/b (ROC AUC 0.75, p-value 0.009). NS, Not Statistically Significant.

To better understand the implications of N-glycans in the context of breast cancer risk, analysis was done on breast cancer progression TMAs that range from hyperplasia, benign and inflammation to metastatic lymph nodes (**Supplementary Figure 9A**). N-glycan peak intensity patterns were assessed in the context of breast cancer progression and differential peak intensities can be observed in a hierarchical clustering heat map (**Supplementary Figure 9b**). Specific N-glycan peaks show distinct intensity patterns based on breast cancer progression TMAs (**Supplementary Figure 10**). More specifically, N-glycan 2012 m/z appears to have a higher peak intensity in the non-malignant TMAs [Normal, Hyperplasia, Benign, Inflammation, Normal Adjacent to the Tumor (NAT) and Adjacent to the Tumor (AT)] relative to the non-metastatic malignant TMAs (Stage 0 – III) (**Figure 5A**). When statistically comparing the peak intensities of N-glycan 2012 m/z, NAT cores had significantly higher intensity compared to non-metastatic malignant cores (p-value <0.01) (**Figure 5B**). ROC curve analysis for peak 2012 m/z comparing the breast cancer stages I-III to NAT shows significant ROC AUC values when comparing NAT vs. Stage I (AUC 0.84; p-value: 0.0192), NAT vs. Stage II (AUC 0.82; p-value: 0.005) and NAT vs. Stage III (AUC 0.84; p-value: 0.009) (**Figure 5C**). When comparing the peak intensity for 2012 m/z between malignant breast cancer stages, Stage 0 had significantly higher peak intensity compared to Stage II and Stage III (p-value <0.01) (**Figure 5D**). No significant difference was observed between Stage 0 and Stage I for 2012 m/z peak intensity. ROC curve analysis for peak 2012 m/z comparing the breast cancer stages 0-III show only significant ROC AUC values when comparing Stage 0 vs. Stage II (AUC 0.71; p-value: 0.007) and Stage 0 vs. Stage III (AUC 0.75; p-value: 0.009) (**Figure 5E**). When comparing Stage I vs. II, Stage I vs. III and Stage II vs. III, the ROC AUC values were not significant (**Supplementary Figure 11**). Similar peak intensity comparison and ROC curve analysis were done for N-glycans 1866 m/z (**Supplementary Figure 12**) and 2158 m/z (**Supplementary Figure 13**).

Interestingly, in addition to high intensity in non-malignant cores, the high 2012 m/z peak intensity is returned in the metastatic lymph TMAs (**Figure 5A**). Comparative analysis reveals that 1866 m/z, 2012 m/z and 2158 m/z have distinctive patterns in metastatic lymph node TMAs (**Supplementary Figure 14**). All three N-glycans were upregulated in metastatic lymph cores relative to non-metastatic malignant and non-malignant cores (**Supplementary Figures 14 A–C**). More specifically, peaks 1866 m/z and 2012 m/z showed significant upregulation of peak intensity in metastatic lymph cores compared to non-metastatic breast malignant cores, Stages I-III. Peak 2158 m/z, however, showed significant peak intensity elevation compared to the non-malignant benign breast cores in addition to Stages II-III cores. ROC curve analysis was conducted for peaks 1866 m/z, 2012 m/z and 2158 m/z (**Supplementary Figures 14 D–F**). Further analysis must be done to determine if the observed differences are driven by different tissue type, N-glycosylation regulation during cancer progression or due to changes in glycoprotein carriers.

## Discussion

Current U.S. statistics show that Black women have higher rates of overweight/obese body mass indexes (BMIs) relative to white women (82, 83) and this is also shown in our data. BMI disparity is important to note because BMI and weight gain have been recognized as an important risk factor for breast cancer (11, 14, 16, 17, 19, 78, 84–86). One study found that a 5 kg/m^2^ increase in BMI corresponded to a 2% increase in breast cancer risk (78). Specifically in BW, obesity has been linked to increased incidence of a specific and more aggressive type of breast cancer, triple negative breast cancer (TNBC) (11, 80, 87). Black women not only experience an unequal burden of obesity (13, 17), but are diagnosed with breast cancer at younger ages and at higher rates of aggressive breast cancer types relative to WW (2, 4, 5, 11).

In the current study, we report that certain glycans show lower ancestry-dependent expression levels in normal breast within the obese groups when stratifying by BMI. We further detected a specific singly-fucosylated N-glycan structure (m/z = 2012) decreased in BW. We showed that in breast cancer progression, the same specific singly-fucosylated N-glycan structure (m/z = 2012) was decreased in malignant breast cores (stages I-III) compared to non-malignant breast samples (hyperplasia, inflammation, normal adjacent and normal). This suggests that glycosylation patterns from WW follow non-malignant breast tissue patterns, while BW follow glycosylation patterns more similar to tumor. Differences in baseline glycosylation in normal breast tissue may be contributing to specific differences in breast stromal biology disproportionally affecting BW. While previous studies have shown that increased fucosylation is associated with malignancy (38, 41, 88–90), one study found that a specific fucosylated glycan structure had been shown to be relatively higher in normal breast compared to breast tumors, both from breast cancer patients (91). Based on the previous findings and our current study, it may not be appropriate to generalize fucosylation patterns in breast tissue as distinct fucosylated structures may have distinct associations in normal versus malignant tissue. To our knowledge, this is the first study to look at the specific fucosylation structures (e.g. 2012 m/z) in normal breast tissue from women without cancer. Thus, the fucosylated N-glycan 2012 m/z observed here may be more closely associated with non-malignant breast tissue relative to malignant breast cancer. Overall, specific ancestry-dependent glycosylation patterns in BW normal breast tissue fall within glycosylation trends detected in early to late breast cancer progression.

Fucosylation is a well-known glycosylation modification that has been well characterized in cancers (32, 35, 38, 48, 49, 92, 93). Variations in fucosylation linkages such as outer-arm fucosylation and core-fucosylation have distinct implications for cancer biology (27, 28, 32, 35, 38, 42, 92). In fact, increased fucosylation, both core and branched segments, have been observed in malignant transformation in many cancers such as breast (38, 41), liver (66), pancreatic (94), prostate (61) and colorectal cancers (95). One study reported a single core-fucosylated polylactosamine glycan was associated with poor clinical outcomes in breast cancer (38). Further, epithelial to mesenchymal transformation (EMT) relies on core-fucosylation (88, 90, 96). In normal tissue converting to malignancies, it is possible that the pattern of emergence involves changes from branched to core fucosylation. There are very few reports on glycosylation in normal breast and breast tissue at risk for cancer. However, a serum study reported that higher BMI was associated with increases in branching and outer-arm fucosylation; and that decreases in core-fucosylation were detected in normal breast and breast cancer patients with increasing BMI (49). Additionally, the same study found that higher mammographic density was associated with decreases in outer-arm fucosylated tri- and tetra-antennary glycans (49). Mammographic density and BMI, both important breast cancer risk factors (49, 84, 97, 98), were observed to have inverse trends of outer-arm fucosylation (49). Studies looking at the relationship between BMI and mammographic density have shown conflicting results (77, 84, 99); however, one study found that the association between mammographic density and breast cancer risk appeared to be the strongest in obese African American women relative to other ancestries (77).

It is important to note that the current data does not determine whether it is the breast epithelium or the stroma driving the observed glycosylation patterns as the sampling strategy did not allow for cellular annotation. Our current studies on normal breast at risk focus on identifying the cellular source of these N-glycans to assess their functional roles in the normal breast tissue microenvironment. Because the samples are stroma rich TMAs, it is possible that specific stroma composition may play a role in breast cancer outcomes that is additionally altered by BMI status. For instance, obesity has been linked to an increased incidence of TNBC in premenopausal and postmenopausal African American women (80). Further, changes in BMI are associated with both metabolic and immune response pathways (100) and immune profiles have been seen to differ by ancestry (101). Thus, the differences in fucosylation observed in WW relative to BW with high risk of breast cancer may contribute to the disparity in tumor aggressiveness. Additionally, changes in N-linked glycoproteins such as HER2 receptors (43) and epidermal growth factor receptors (EGFR) (89) that have been observed in breast cancer progression could be differentially glycosylated between BW and WW. Based on current literature and our findings, it is possible that in normal tissue, changes in fucose patterns may be contributing to a tumor-permissive microenvironment associated with more severe breast cancers influenced by genetic ancestry and BMI. A limitation of this exploratory study on BMI is that multiple comparisons among the limited number of women may increase the false discovery rate of the findings. Work is in progress to investigate ancestry-dependent patterns of core versus outer-arm fucosylated N-glycans in normal breast tissue with high risk of breast cancer.

Finally, our initial studies using linear regression data revealed distinct N-glycans associated with specific socioeconomic factors by ancestry. Previous literature supports the link between socioeconomic factors and their effects on health (102–107). One study found that maternal education level, compared to paternal education level, may be less important in influencing the risk of low birthweights, a positive association for breast cancer risk (108), in black families (106). Additionally, childhood socioeconomic status may also contribute to health outcome in adulthood (102) implying intergenerational socioeconomic stresses can contribute to epigenetic modifications and poor health. The relationship between socioeconomic stresses and health impact is not a novel concept; generally, distinct biological alterations could be considered to be a reflection of different socioeconomic conditions (109–111). However, the affect socioeconomic status has on specific biological factors, processes and modifications contributing to development of disease such as breast cancer is poorly understood. Our results suggest that from a subset of N-glycans significantly altered by ancestry, certain N-glycans were strongly associated with household income for WW, while the same N-glycans were strongly associated with marital status for BW. This may imply that immune responses triggered by specific lifestyle stressors are different throughout WW and BW; future studies should look at glycosylation changes associated with immune components. This research is promising towards linking molecular markers to socioeconomic stress. However, more research needs to be done to identify specific N-glycan biomarkers associated with socioeconomic stresses like household income and marriage in order to determine which glycan signatures are associated with increased breast cancer risk. This broadens our understanding of glycosylation regulation and the possibility that such regulation may be influenced by socioeconomic stressors. To our knowledge, this is the first study to investigate N-glycan patterns associated with socioeconomic stresses that may differ by ancestry. This is important because it contributes to the understanding of the complexities linking socioeconomic stresses and molecular factors to breast cancer risk and aggressiveness in black women. Additional factors to consider include family size, childhood socioeconomic status, and educational quality as these are factors that can also play a role in the ongoing health disparities BW face.

In summary, breast cancer is now the leading cause of cancer death among women and disproportionately affects BW at significantly higher rates than WW. Thus, there is great need to close this gap and lower the overall breast cancer related death rates. Improving breast cancer prognosis and death rates requires a research focus on cancer control and prevention. Our study focused on ancestry-dependent glycosylation patterns in normal breast tissues from women at risk for breast cancer. We demonstrated that unique N-glycosylation patterns in normal breast tissues can differentiate BW from WW with high risk of breast cancer. More specifically, we revealed that in tissue at risk for breast cancer, decreases in specific fucosylated glycans in BW relative to WW may contribute to differences in breast stroma biology that could account in part for differences in breast cancer subtypes. Future studies should aim to investigate cell-specific N-glycan molecular signatures in breast tissue and immune cells and their roles in creating a tumor-permissive tissue microenvironment.

## Data Availability Statement

The original contributions presented in the study are included in the article/supplementary materials, further inquiries can be directed to the corresponding author/s.

## Ethics Statement

The studies involving human participants were reviewed and approved by exemption 4 status by the Medical University of South Carolina Institutional Review Board. Written informed consent for participation was not required for this study in accordance with the national legislation and the institutional requirements.

## Author Contributions

Conceptualization, DR, DS, and PA; methodology, DR, DS, and PA; software, DR, DS, PA; validation, DR and PA; formal analysis, DR and PA; investigation, DR and PA; resources, HN, GS, RD, AM, and PA; data curation, DR, and PA; writing – original draft preparation, DR and PA; writing – review and editing, DR, PA, RD, YP, DS, and HN; visualization, DR and PA; supervision, PA; project administration, PA; funding acquisition PA. All authors have read and agreed to the published version of the manuscript.

## Funding

PA appreciates support by NIH/NCI R21 CA240148, R01 CA253460 and in part by pilot research funding from the Hollings Cancer Center Support Grant from NIH/NCI P30 CA138313 at the Medical University of South Carolina. DR is grateful for support from NIH/NIGMS R25GM072643. Additional support to RD provided by the South Carolina Centers of Economic Excellence SmartState program. The MUSC Imaging Mass Spectrometry Research Resource is supported in part by the NIH/NIDDK Digestive Disease Research Core Center P30DK123704 and is a division of the Mass Spectrometry Facility is supported by the University and P20GM103542 (NIH/NIGMS).

## Conflict of Interest

The authors declare that the research was conducted in the absence of any commercial or financial relationships that could be construed as a potential conflict of interest.

## Publisher’s Note

All claims expressed in this article are solely those of the authors and do not necessarily represent those of their affiliated organizations, or those of the publisher, the editors and the reviewers. Any product that may be evaluated in this article, or claim that may be made by its manufacturer, is not guaranteed or endorsed by the publisher.
