# Supplementary material for: Metabolic Links to Socioeconomic Stresses Uniquely Affecting Ancestry in Normal Breast Tissue at Risk for Breast Cancer
Source: Front Oncol. 2022 Jun 27;12:876651. doi: 10.3389/fonc.2022.876651 (PMC9273232; doi:10.3389/fonc.2022.876651)
Supplement: Supplementary file 1 [file Presentation_1.pptx]

## Slide 1
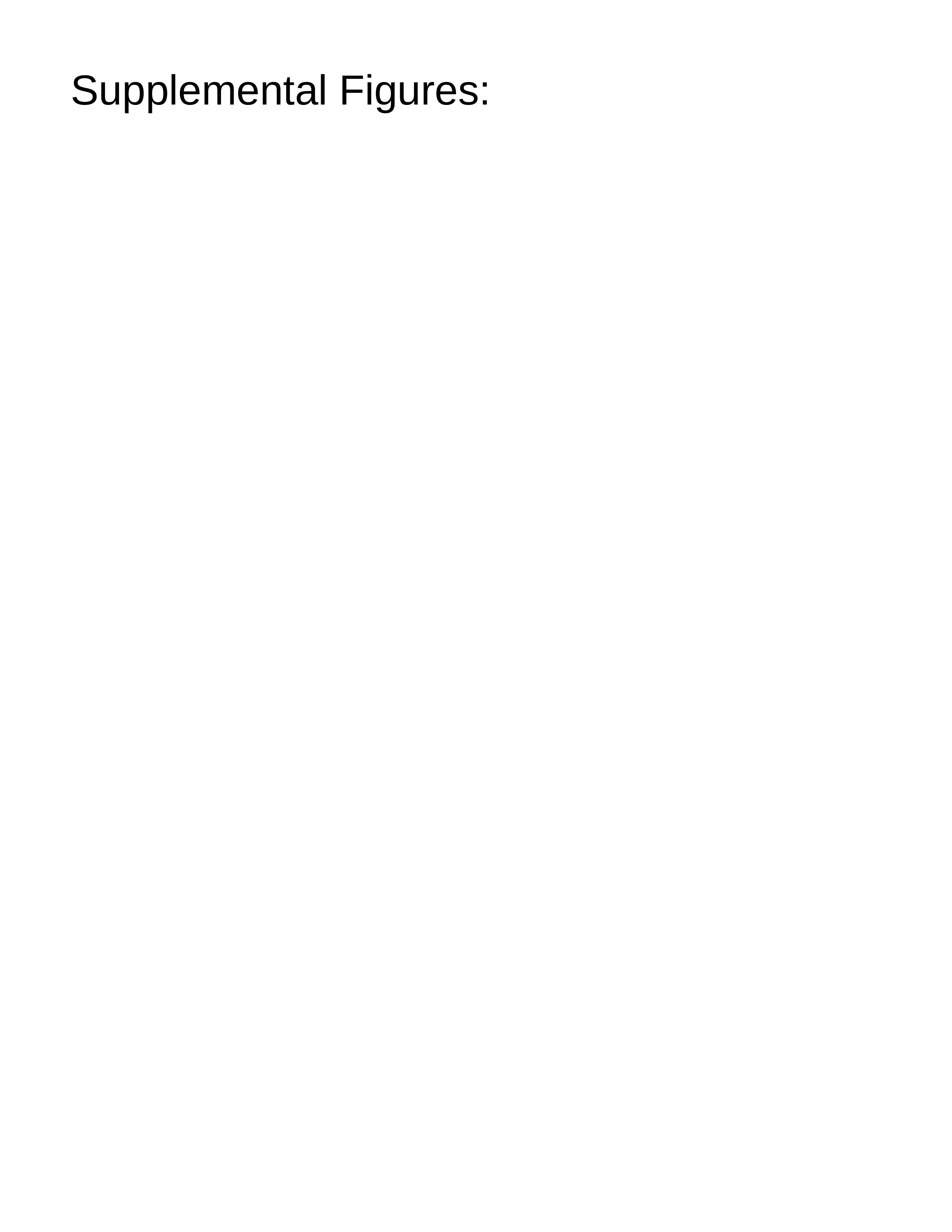

Supplemental Figures:

## Slide 2
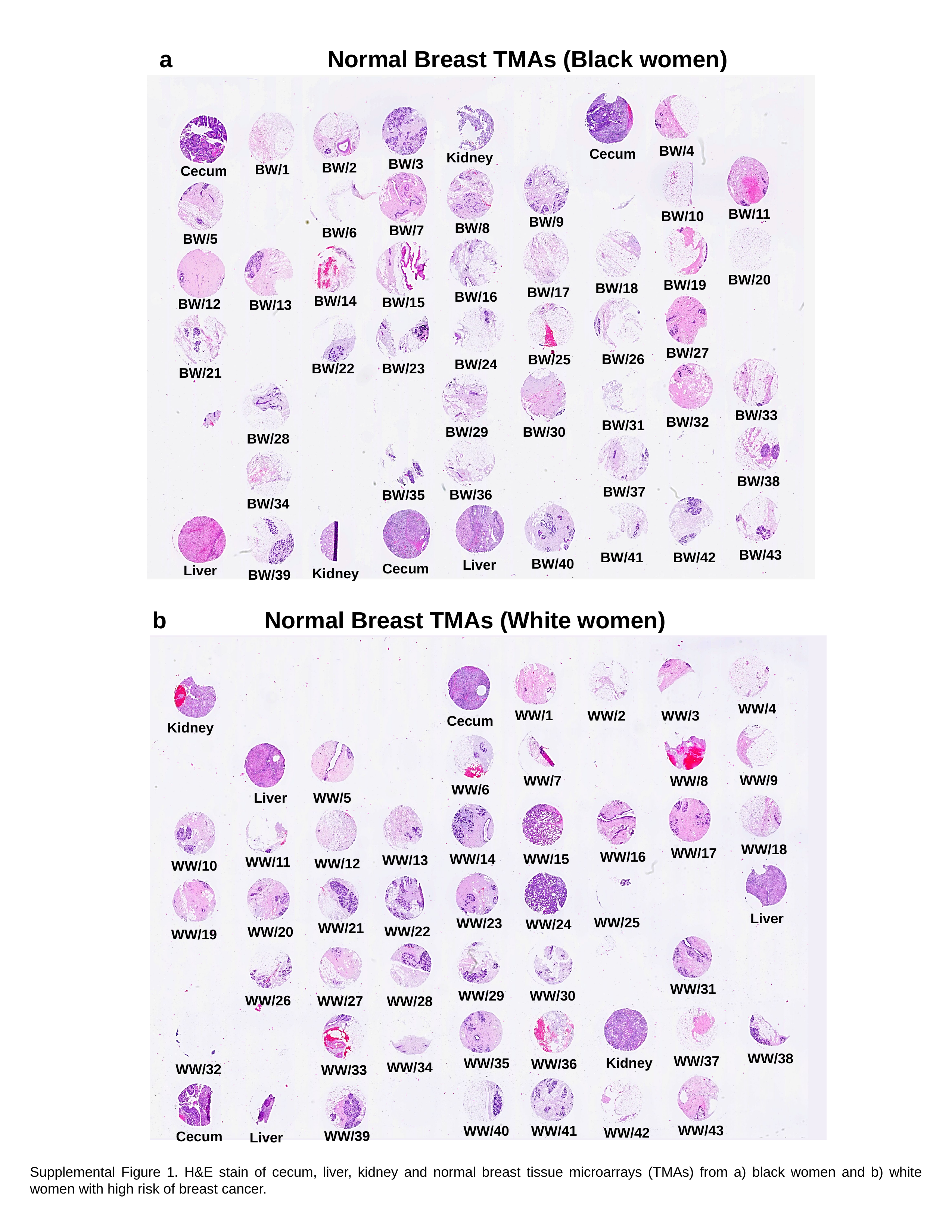

a 			Normal Breast TMAs (Black women)
Cecum
BW/4
Kidney
BW/3
BW/2
BW/1
Cecum
BW/11
BW/10
BW/9
BW/8
BW/7
BW/6
BW/5
BW/20
BW/19
BW/18
BW/17
BW/16
BW/14
BW/15
BW/12
BW/13
BW/27
BW/26
BW/25
BW/24
BW/22
BW/23
BW/21
BW/33
BW/32
BW/31
BW/29
BW/30
BW/28
BW/38
BW/37
BW/36
BW/35
BW/34
BW/43
BW/41
BW/42
BW/40
Liver
Cecum
Liver
Kidney
BW/39
b 		Normal Breast TMAs (White women)
WW/4
WW/1
WW/2
WW/3
Cecum
Kidney
WW/9
WW/7
WW/8
WW/6
WW/5
Liver
WW/18
WW/17
WW/16
WW/15
WW/14
WW/13
WW/11
WW/12
WW/10
Liver
WW/25
WW/23
WW/24
WW/21
WW/20
WW/22
WW/19
WW/31
WW/30
WW/29
WW/26
WW/27
WW/28
WW/38
WW/37
Kidney
WW/35
WW/36
WW/34
WW/32
WW/33
WW/41
WW/43
WW/40
WW/42
WW/39
Cecum
Liver
Supplemental Figure 1. H&E stain of cecum, liver, kidney and normal breast tissue microarrays (TMAs) from a) black women and b) white women with high risk of breast cancer.

## Slide 3
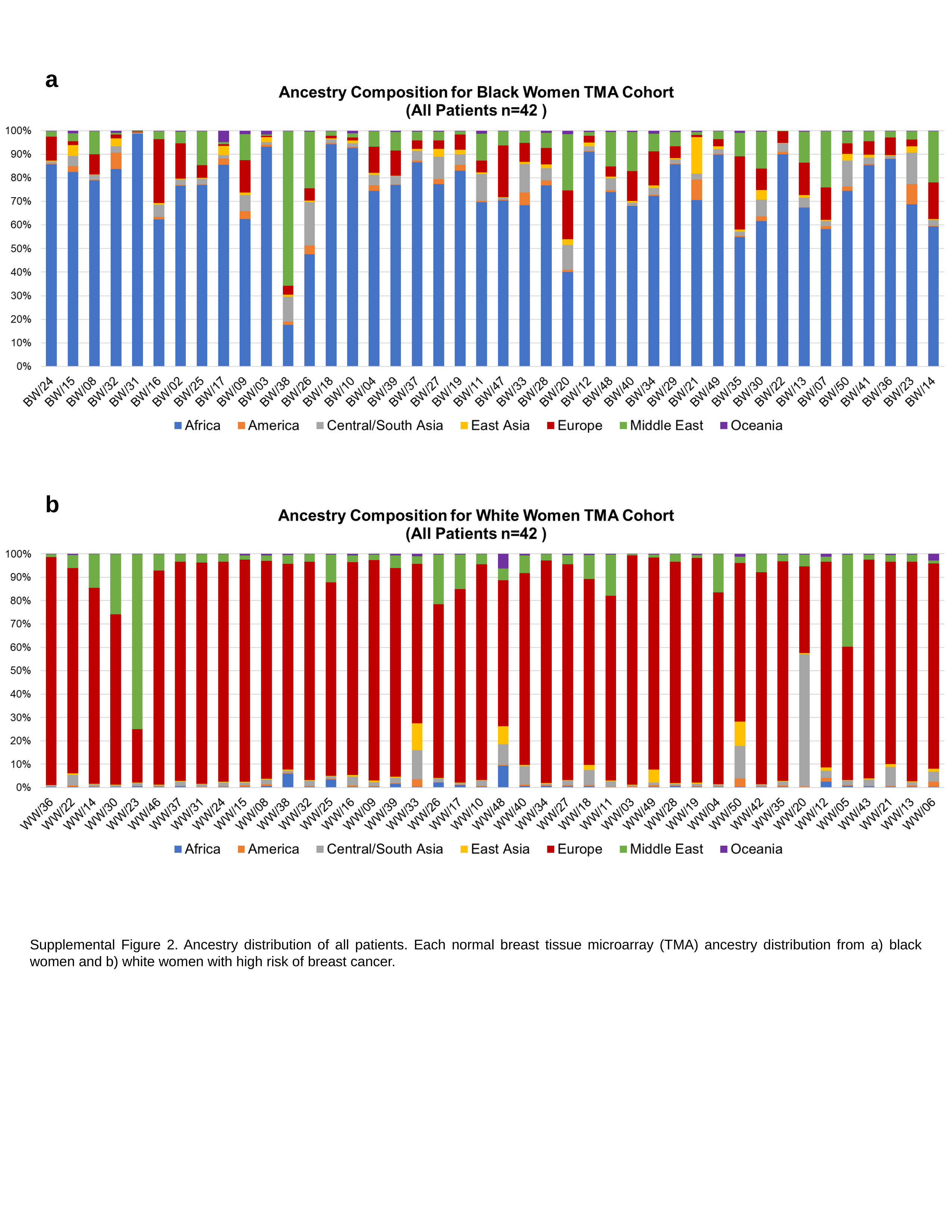

a
b
Supplemental Figure 2. Ancestry distribution of all patients. Each normal breast tissue microarray (TMA) ancestry distribution from a) black women and b) white women with high risk of breast cancer.

## Slide 4
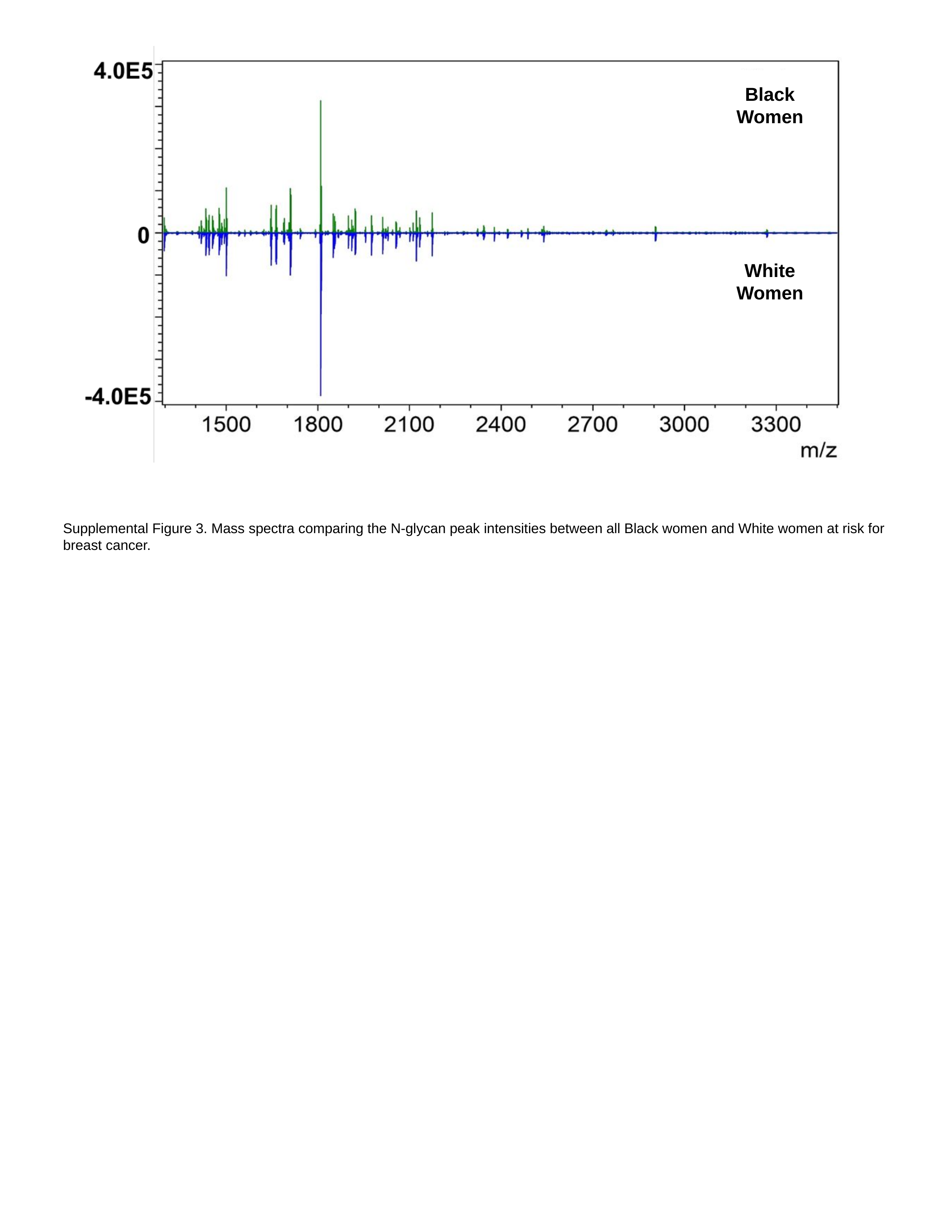

Black Women
White Women
Supplemental Figure 3. Mass spectra comparing the N-glycan peak intensities between all Black women and White women at risk for breast cancer.

## Slide 5
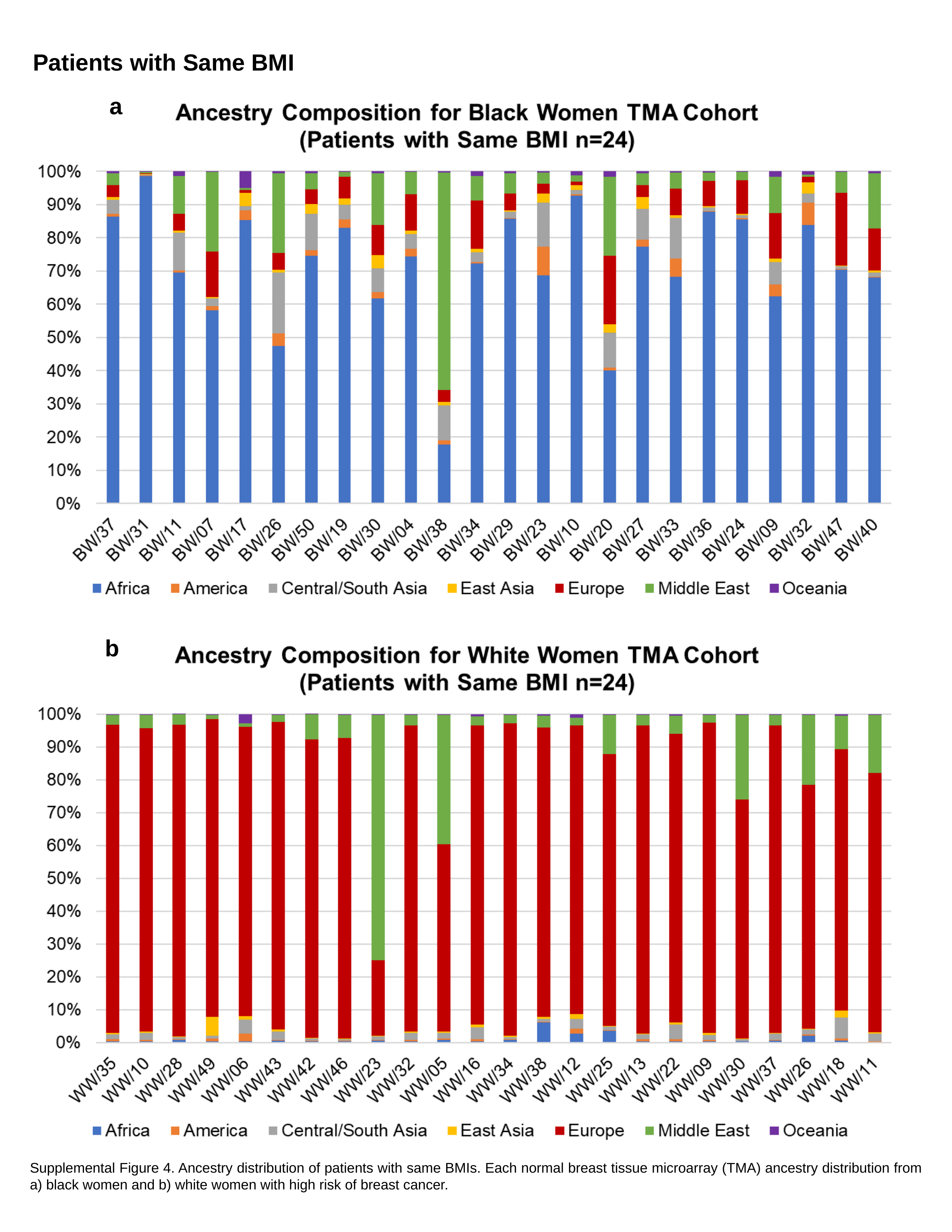

Patients with Same BMI
a
b
Supplemental Figure 4. Ancestry distribution of patients with same BMIs. Each normal breast tissue microarray (TMA) ancestry distribution from a) black women and b) white women with high risk of breast cancer.

## Slide 6
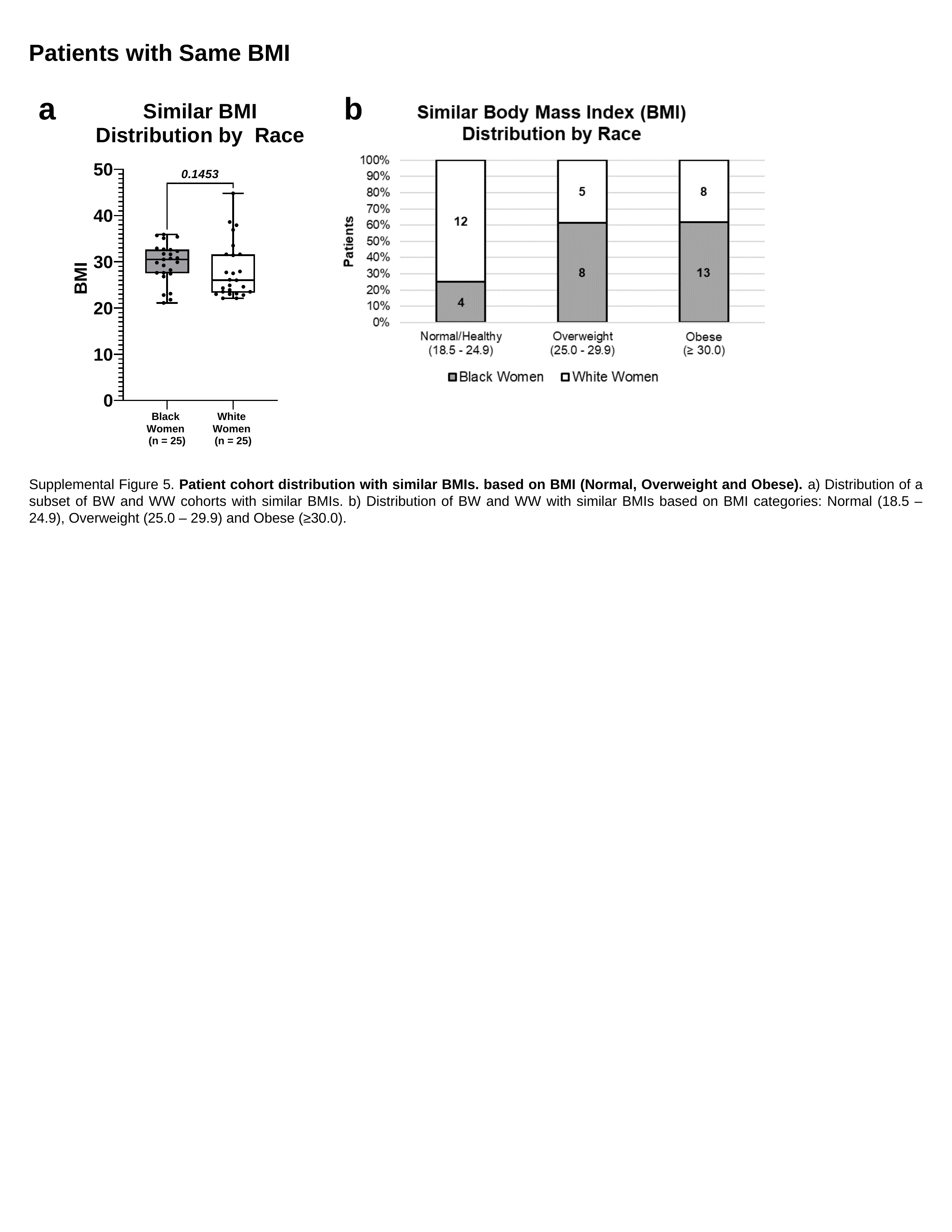

Patients with Same BMI
a
b
Supplemental Figure 5. Patient cohort distribution with similar BMIs. based on BMI (Normal, Overweight and Obese). a) Distribution of a subset of BW and WW cohorts with similar BMIs. b) Distribution of BW and WW with similar BMIs based on BMI categories: Normal (18.5 – 24.9), Overweight (25.0 – 29.9) and Obese (≥30.0).

## Slide 7
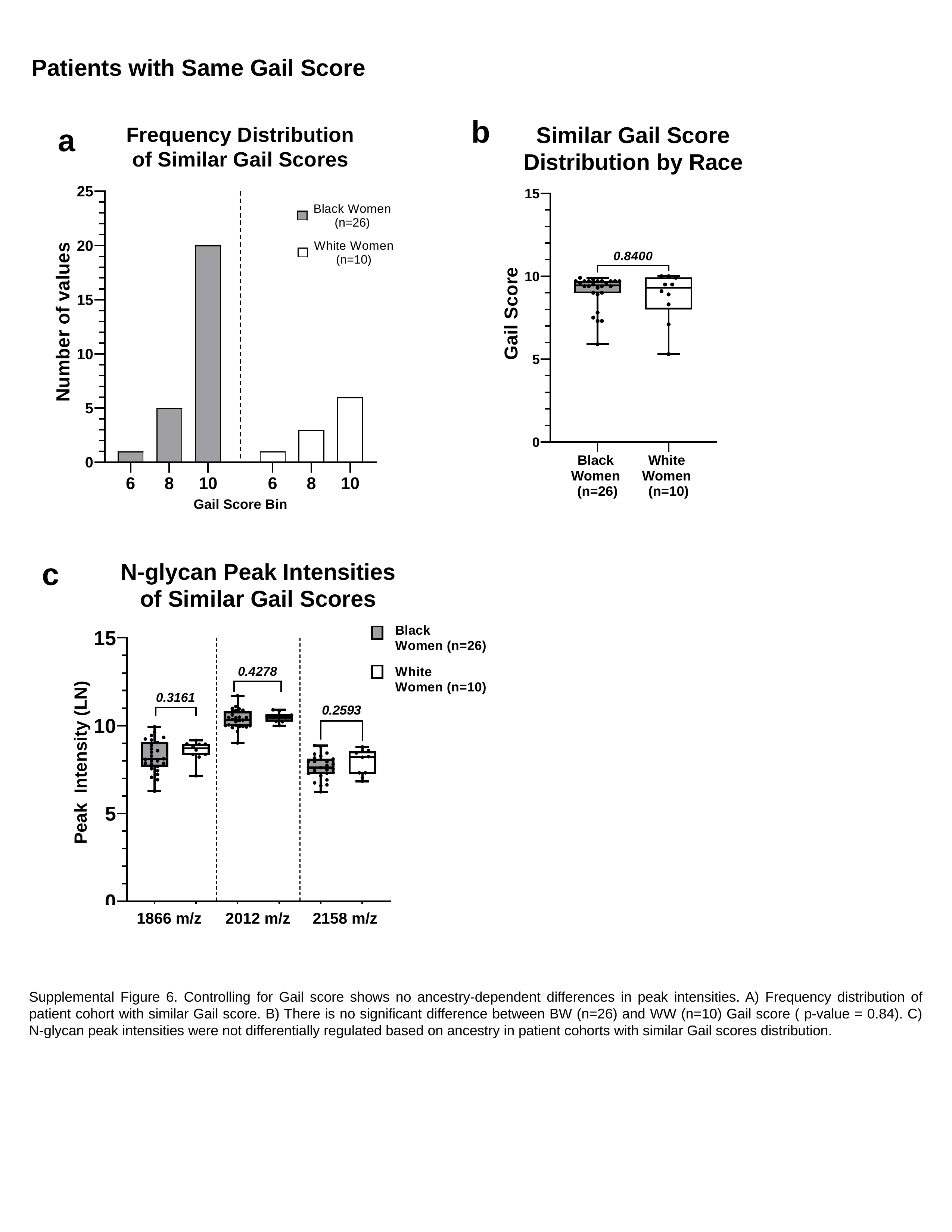

Patients with Same Gail Score
b
a
c
1866 m/z
2012 m/z
2158 m/z
Supplemental Figure 6. Controlling for Gail score shows no ancestry-dependent differences in peak intensities. A) Frequency distribution of patient cohort with similar Gail score. B) There is no significant difference between BW (n=26) and WW (n=10) Gail score ( p-value = 0.84). C) N-glycan peak intensities were not differentially regulated based on ancestry in patient cohorts with similar Gail scores distribution.

## Slide 8
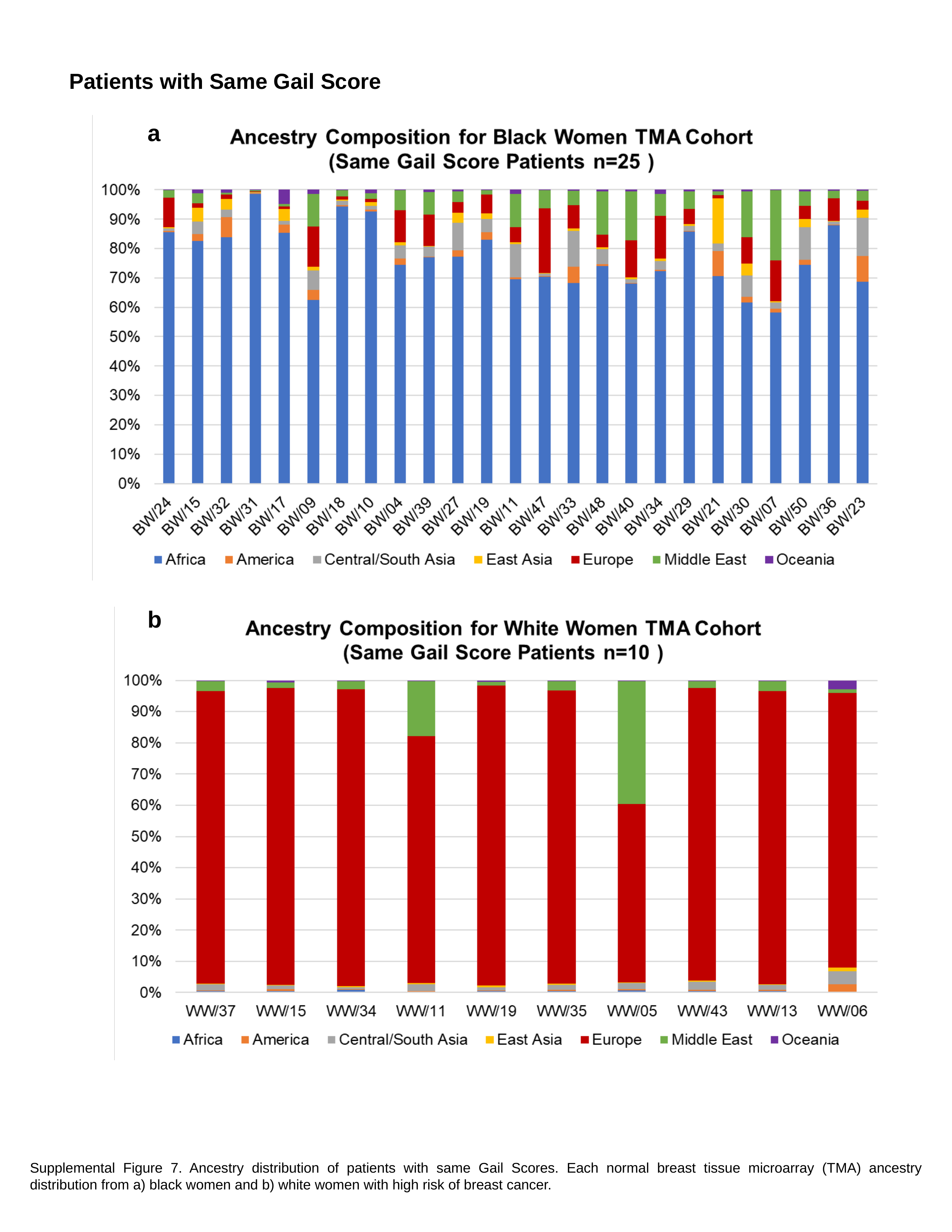

Patients with Same Gail Score
a
b
Supplemental Figure 7. Ancestry distribution of patients with same Gail Scores. Each normal breast tissue microarray (TMA) ancestry distribution from a) black women and b) white women with high risk of breast cancer.

## Slide 9
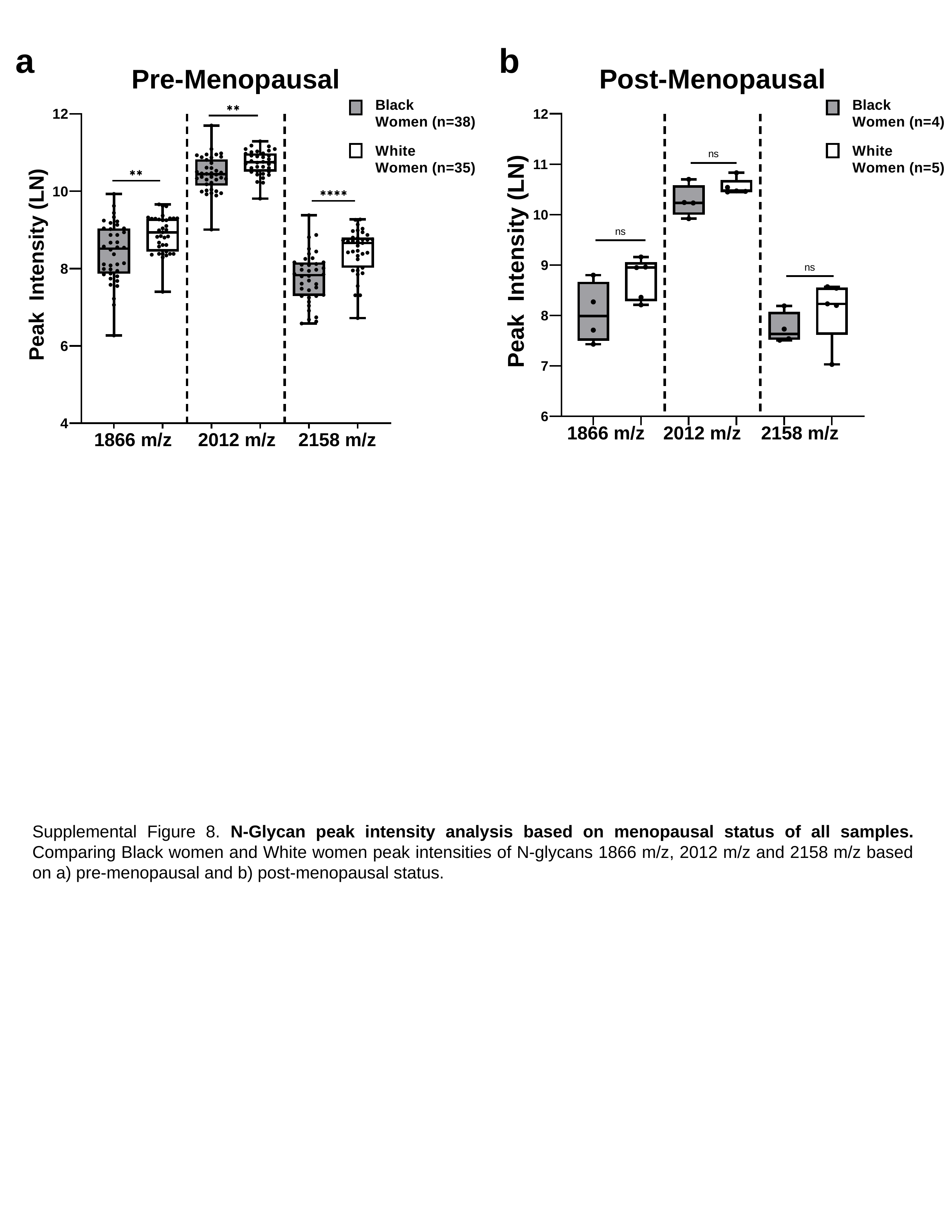

a
b
1866 m/z
2012 m/z
2158 m/z
1866 m/z
2012 m/z
2158 m/z
Supplemental Figure 8. N-Glycan peak intensity analysis based on menopausal status of all samples. Comparing Black women and White women peak intensities of N-glycans 1866 m/z, 2012 m/z and 2158 m/z based on a) pre-menopausal and b) post-menopausal status.

## Slide 10
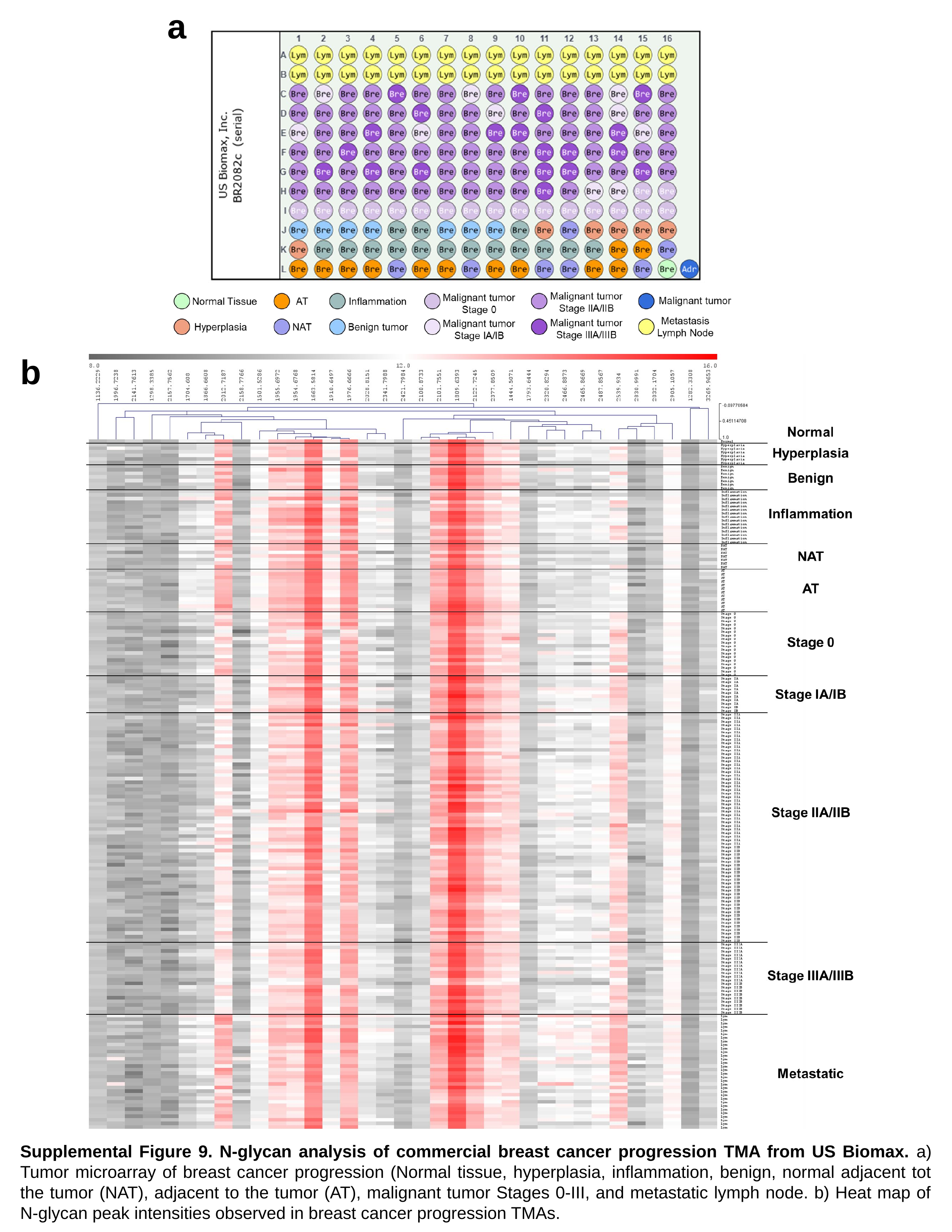

a
b
Supplemental Figure 9. N-glycan analysis of commercial breast cancer progression TMA from US Biomax. a) Tumor microarray of breast cancer progression (Normal tissue, hyperplasia, inflammation, benign, normal adjacent tot the tumor (NAT), adjacent to the tumor (AT), malignant tumor Stages 0-III, and metastatic lymph node. b) Heat map of N-glycan peak intensities observed in breast cancer progression TMAs.

## Slide 11
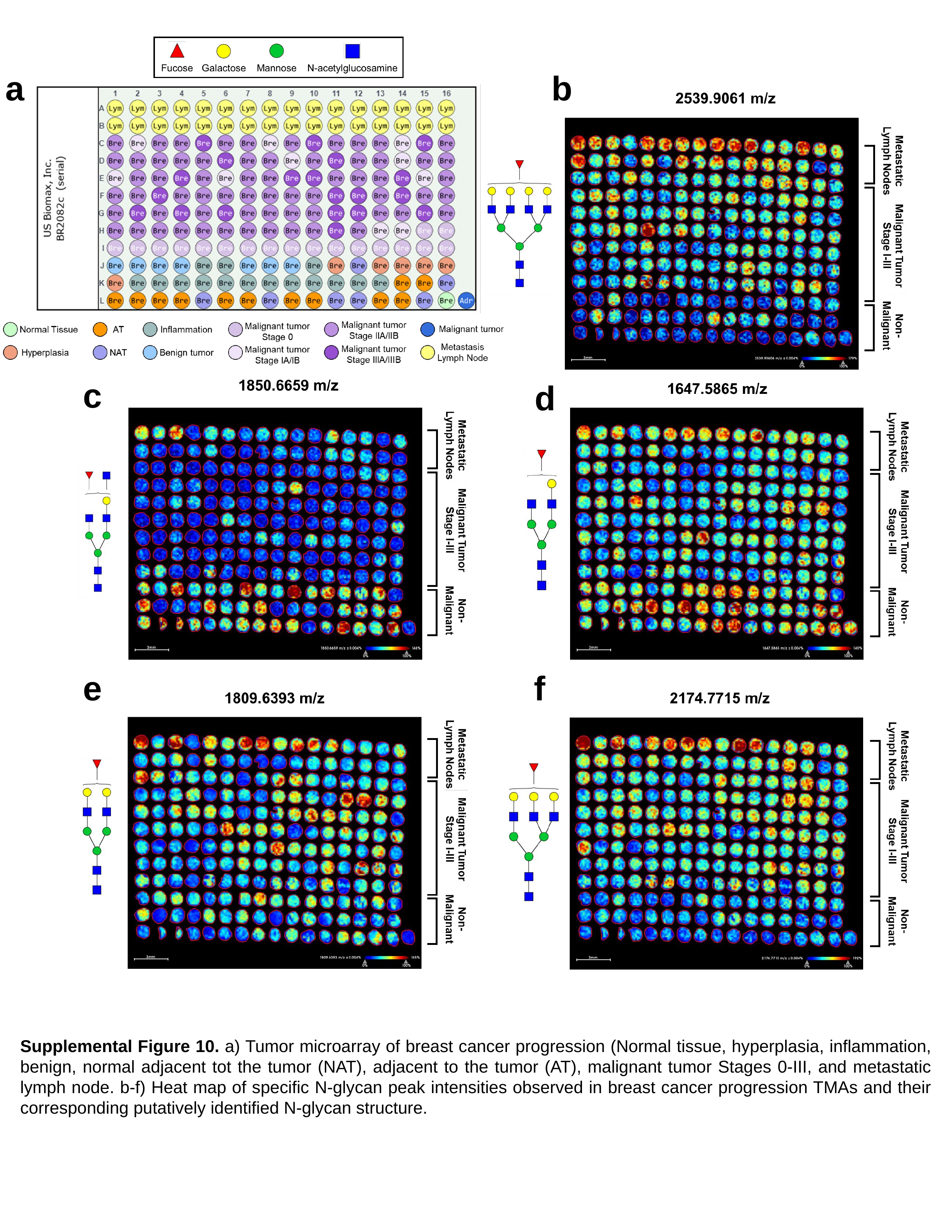

a
b
c
d
e
f
Supplemental Figure 10. a) Tumor microarray of breast cancer progression (Normal tissue, hyperplasia, inflammation, benign, normal adjacent tot the tumor (NAT), adjacent to the tumor (AT), malignant tumor Stages 0-III, and metastatic lymph node. b-f) Heat map of specific N-glycan peak intensities observed in breast cancer progression TMAs and their corresponding putatively identified N-glycan structure.

## Slide 12
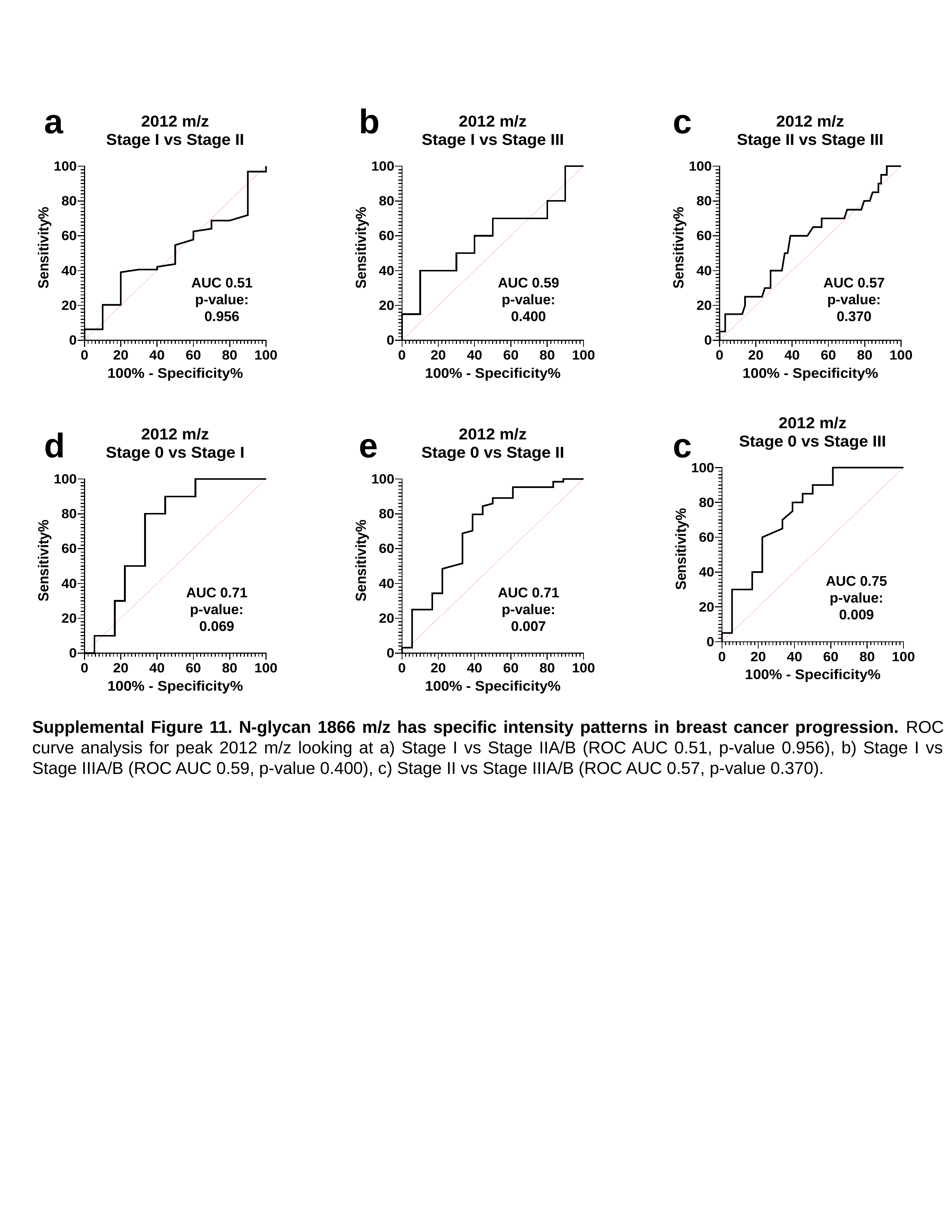

a
b
c
AUC 0.51
p-value: 0.956
AUC 0.59
p-value: 0.400
AUC 0.57
p-value: 0.370
AUC 0.75
p-value: 0.009
d
AUC 0.71
p-value: 0.007
e
c
AUC 0.71
p-value: 0.069
Supplemental Figure 11. N-glycan 1866 m/z has specific intensity patterns in breast cancer progression. ROC curve analysis for peak 2012 m/z looking at a) Stage I vs Stage IIA/B (ROC AUC 0.51, p-value 0.956), b) Stage I vs Stage IIIA/B (ROC AUC 0.59, p-value 0.400), c) Stage II vs Stage IIIA/B (ROC AUC 0.57, p-value 0.370).

## Slide 13
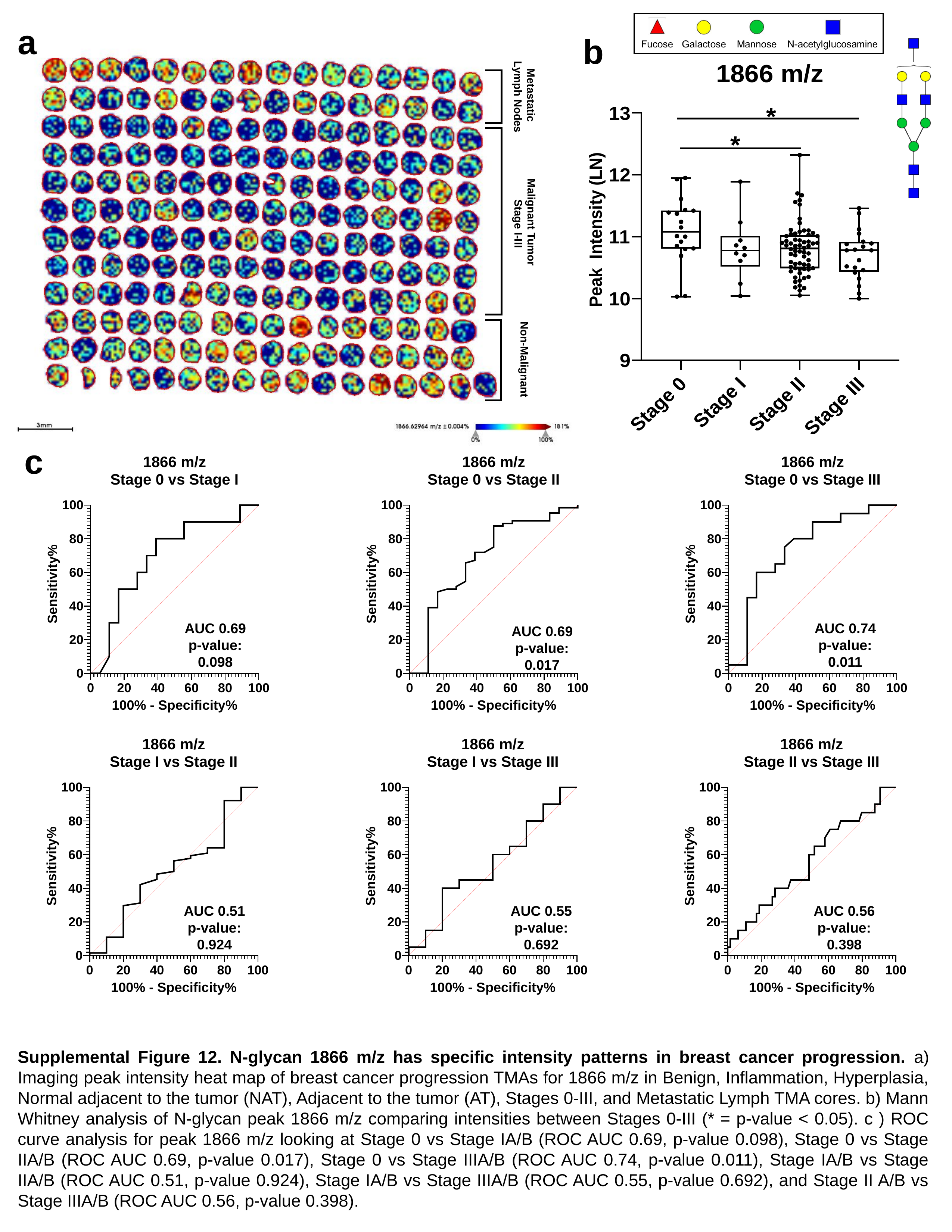

a
b
Metastatic
 Lymph Nodes
Malignant Tumor
Stage I-III
Non-Malignant
c
AUC 0.69
p-value: 0.098
AUC 0.69
p-value: 0.017
AUC 0.74
p-value: 0.011
AUC 0.51
p-value: 0.924
AUC 0.55
p-value: 0.692
AUC 0.56
p-value: 0.398
Supplemental Figure 12. N-glycan 1866 m/z has specific intensity patterns in breast cancer progression. a) Imaging peak intensity heat map of breast cancer progression TMAs for 1866 m/z in Benign, Inflammation, Hyperplasia, Normal adjacent to the tumor (NAT), Adjacent to the tumor (AT), Stages 0-III, and Metastatic Lymph TMA cores. b) Mann Whitney analysis of N-glycan peak 1866 m/z comparing intensities between Stages 0-III (* = p-value < 0.05). c ) ROC curve analysis for peak 1866 m/z looking at Stage 0 vs Stage IA/B (ROC AUC 0.69, p-value 0.098), Stage 0 vs Stage IIA/B (ROC AUC 0.69, p-value 0.017), Stage 0 vs Stage IIIA/B (ROC AUC 0.74, p-value 0.011), Stage IA/B vs Stage IIA/B (ROC AUC 0.51, p-value 0.924), Stage IA/B vs Stage IIIA/B (ROC AUC 0.55, p-value 0.692), and Stage II A/B vs Stage IIIA/B (ROC AUC 0.56, p-value 0.398).

## Slide 14
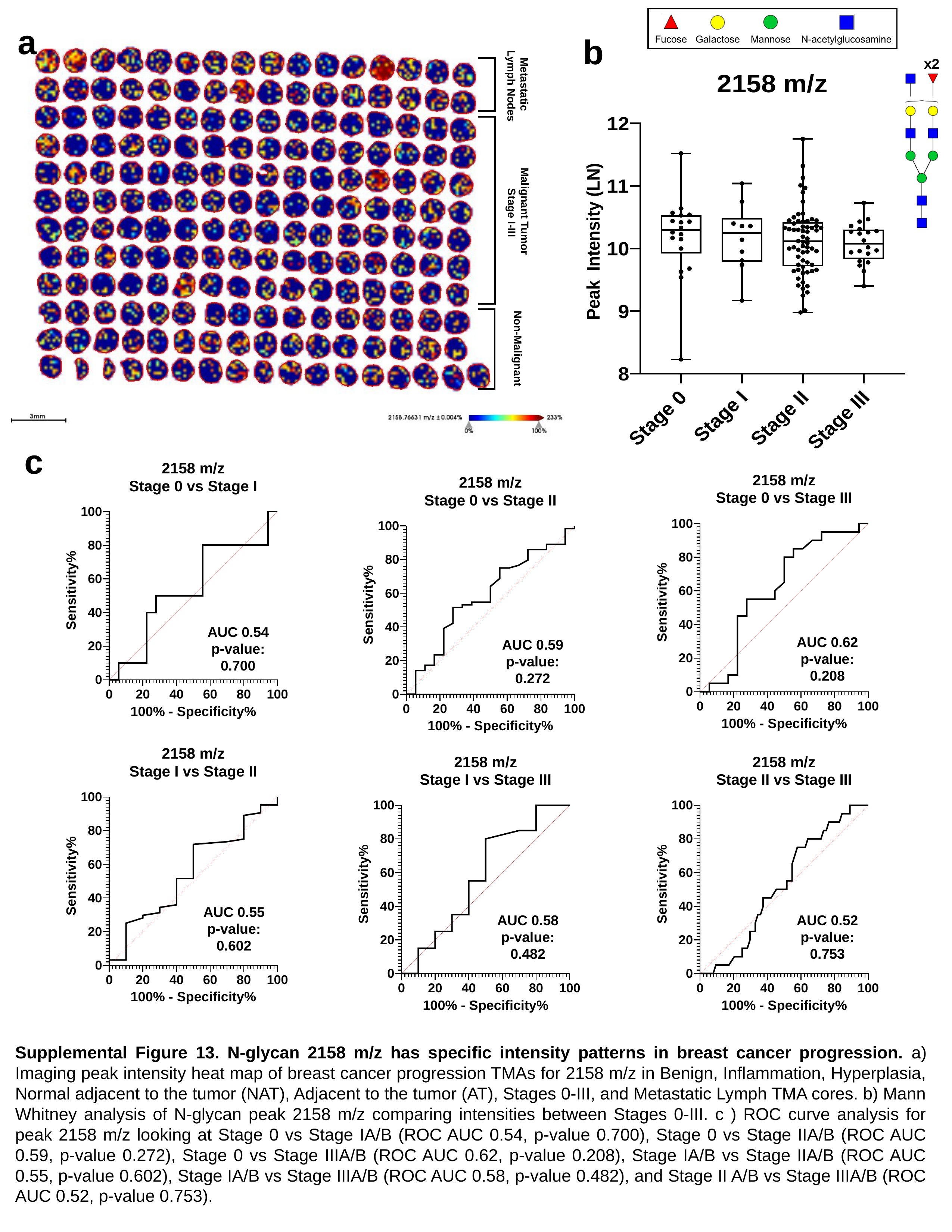

a
Metastatic
 Lymph Nodes
Malignant Tumor
Stage I-III
Non-Malignant
b
x2
c
AUC 0.54
p-value: 0.700
AUC 0.62
p-value: 0.208
AUC 0.59
p-value: 0.272
AUC 0.55
p-value: 0.602
AUC 0.58
p-value: 0.482
AUC 0.52
p-value: 0.753
Supplemental Figure 13. N-glycan 2158 m/z has specific intensity patterns in breast cancer progression. a) Imaging peak intensity heat map of breast cancer progression TMAs for 2158 m/z in Benign, Inflammation, Hyperplasia, Normal adjacent to the tumor (NAT), Adjacent to the tumor (AT), Stages 0-III, and Metastatic Lymph TMA cores. b) Mann Whitney analysis of N-glycan peak 2158 m/z comparing intensities between Stages 0-III. c ) ROC curve analysis for peak 2158 m/z looking at Stage 0 vs Stage IA/B (ROC AUC 0.54, p-value 0.700), Stage 0 vs Stage IIA/B (ROC AUC 0.59, p-value 0.272), Stage 0 vs Stage IIIA/B (ROC AUC 0.62, p-value 0.208), Stage IA/B vs Stage IIA/B (ROC AUC 0.55, p-value 0.602), Stage IA/B vs Stage IIIA/B (ROC AUC 0.58, p-value 0.482), and Stage II A/B vs Stage IIIA/B (ROC AUC 0.52, p-value 0.753).

## Slide 15
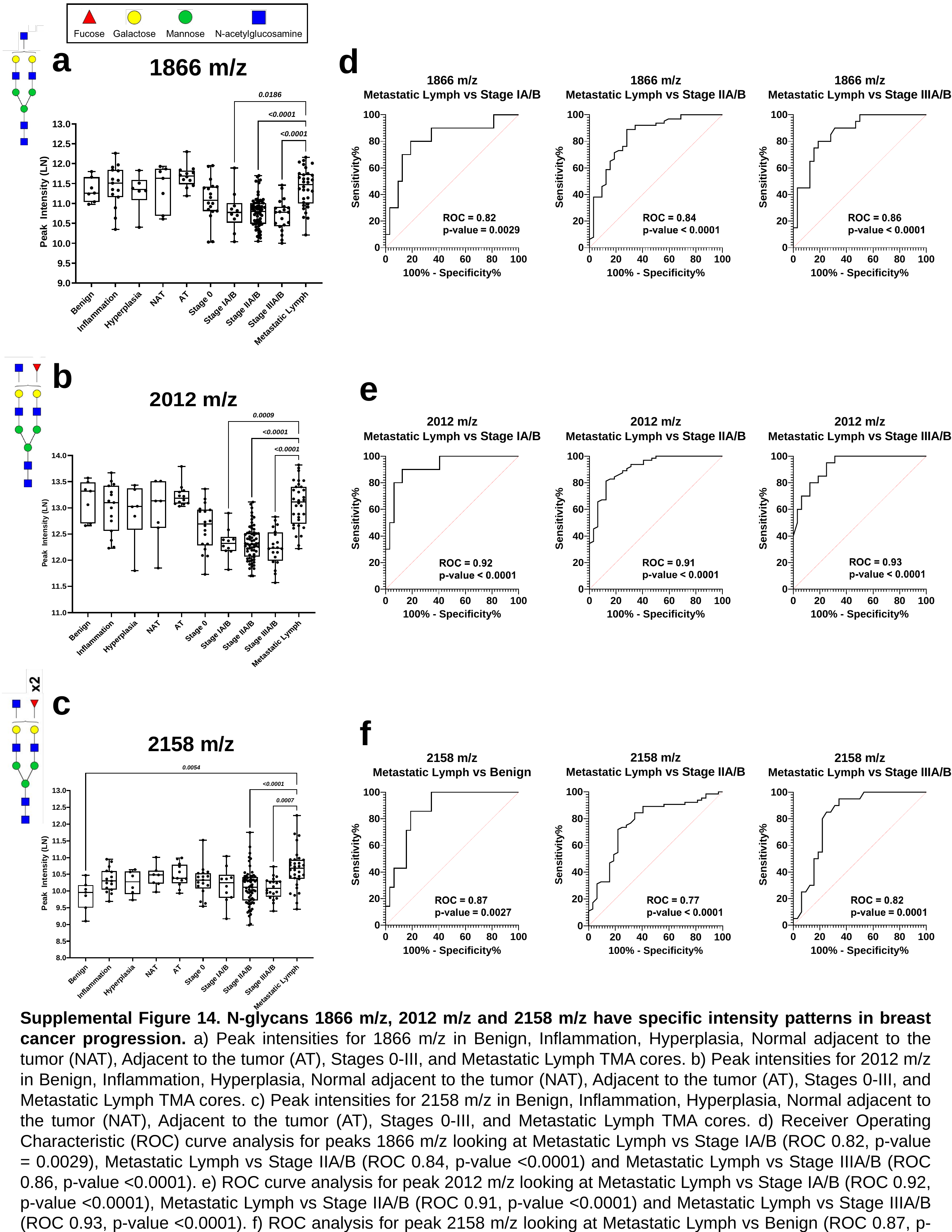

a
d
b
e
c
f
Supplemental Figure 14. N-glycans 1866 m/z, 2012 m/z and 2158 m/z have specific intensity patterns in breast cancer progression. a) Peak intensities for 1866 m/z in Benign, Inflammation, Hyperplasia, Normal adjacent to the tumor (NAT), Adjacent to the tumor (AT), Stages 0-III, and Metastatic Lymph TMA cores. b) Peak intensities for 2012 m/z in Benign, Inflammation, Hyperplasia, Normal adjacent to the tumor (NAT), Adjacent to the tumor (AT), Stages 0-III, and Metastatic Lymph TMA cores. c) Peak intensities for 2158 m/z in Benign, Inflammation, Hyperplasia, Normal adjacent to the tumor (NAT), Adjacent to the tumor (AT), Stages 0-III, and Metastatic Lymph TMA cores. d) Receiver Operating Characteristic (ROC) curve analysis for peaks 1866 m/z looking at Metastatic Lymph vs Stage IA/B (ROC 0.82, p-value = 0.0029), Metastatic Lymph vs Stage IIA/B (ROC 0.84, p-value <0.0001) and Metastatic Lymph vs Stage IIIA/B (ROC 0.86, p-value <0.0001). e) ROC curve analysis for peak 2012 m/z looking at Metastatic Lymph vs Stage IA/B (ROC 0.92, p-value <0.0001), Metastatic Lymph vs Stage IIA/B (ROC 0.91, p-value <0.0001) and Metastatic Lymph vs Stage IIIA/B (ROC 0.93, p-value <0.0001). f) ROC analysis for peak 2158 m/z looking at Metastatic Lymph vs Benign (ROC 0.87, p-value = 0.0027), Metastatic Lymph vs Stage IIA/B (ROC 0.77, p-value <0.0001) and Metastatic Lymph vs Stage IIIA/B (ROC 0.82, p-value = 0.0001).

## Slide 16
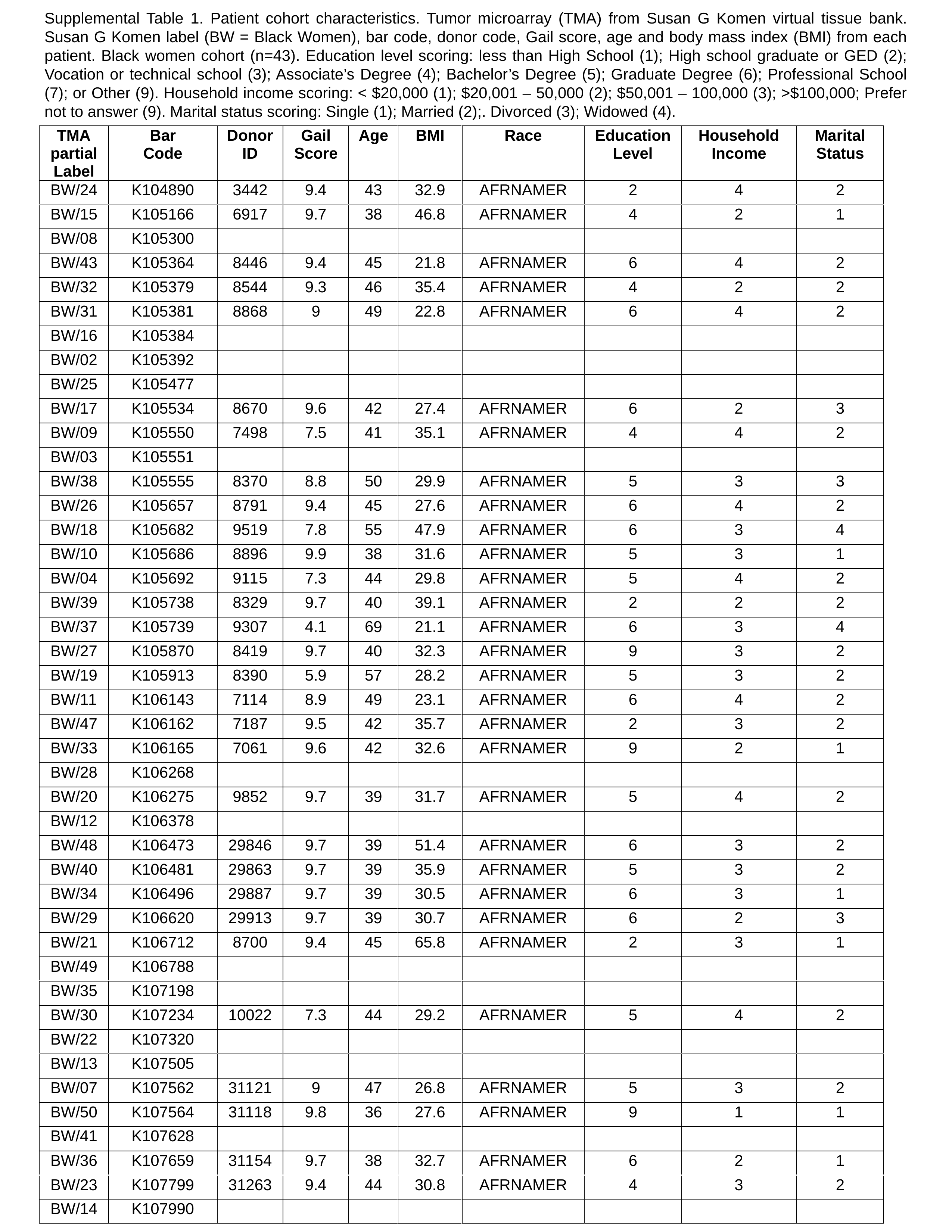

Supplemental Table 1. Patient cohort characteristics. Tumor microarray (TMA) from Susan G Komen virtual tissue bank. Susan G Komen label (BW = Black Women), bar code, donor code, Gail score, age and body mass index (BMI) from each patient. Black women cohort (n=43). Education level scoring: less than High School (1); High school graduate or GED (2); Vocation or technical school (3); Associate’s Degree (4); Bachelor’s Degree (5); Graduate Degree (6); Professional School (7); or Other (9). Household income scoring: < $20,000 (1); $20,001 – 50,000 (2); $50,001 – 100,000 (3); >$100,000; Prefer not to answer (9). Marital status scoring: Single (1); Married (2);. Divorced (3); Widowed (4).

## Slide 17
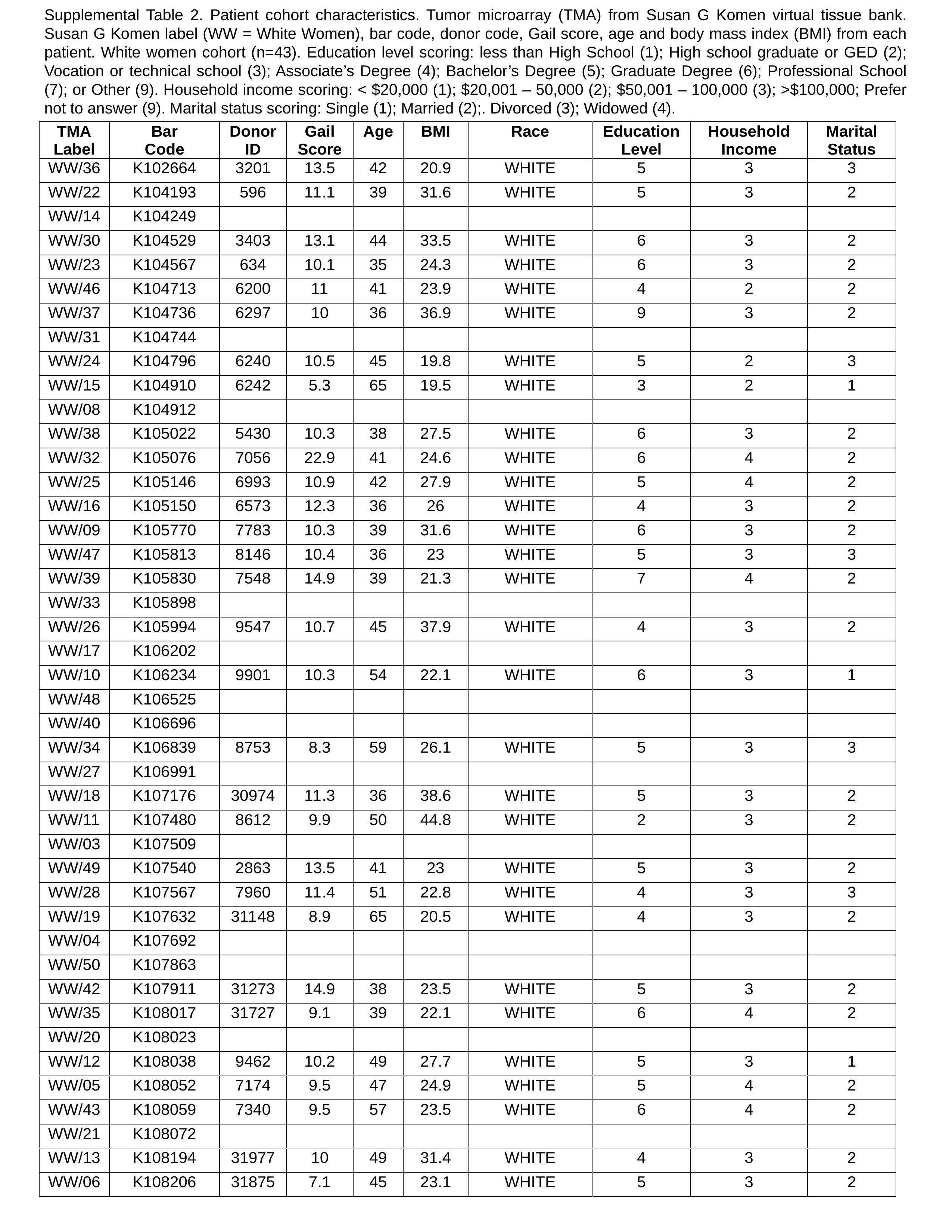

Supplemental Table 2. Patient cohort characteristics. Tumor microarray (TMA) from Susan G Komen virtual tissue bank. Susan G Komen label (WW = White Women), bar code, donor code, Gail score, age and body mass index (BMI) from each patient. White women cohort (n=43). Education level scoring: less than High School (1); High school graduate or GED (2); Vocation or technical school (3); Associate’s Degree (4); Bachelor’s Degree (5); Graduate Degree (6); Professional School (7); or Other (9). Household income scoring: < $20,000 (1); $20,001 – 50,000 (2); $50,001 – 100,000 (3); >$100,000; Prefer not to answer (9). Marital status scoring: Single (1); Married (2);. Divorced (3); Widowed (4).

## Slide 18
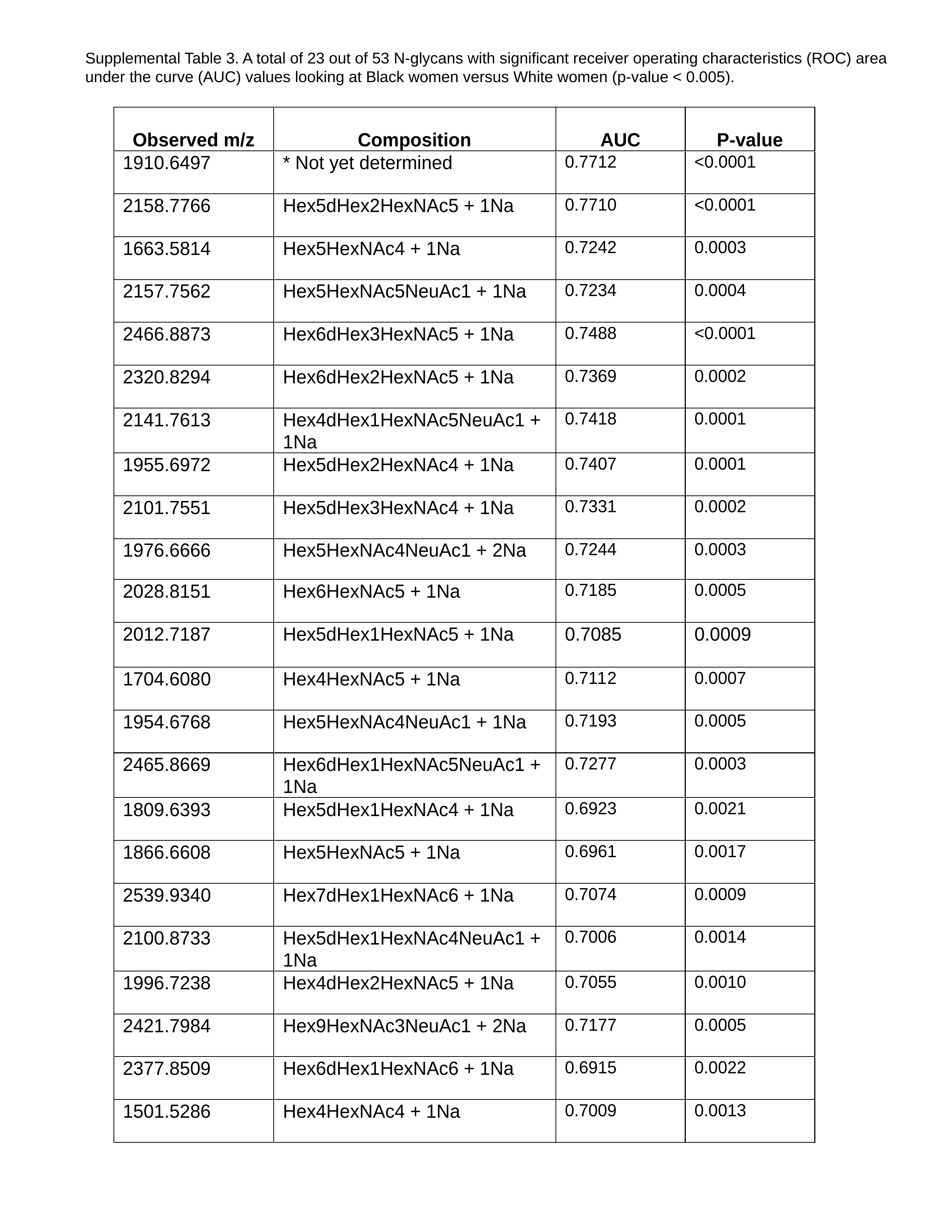

Supplemental Table 3. A total of 23 out of 53 N-glycans with significant receiver operating characteristics (ROC) area under the curve (AUC) values looking at Black women versus White women (p-value < 0.005).

## Slide 19
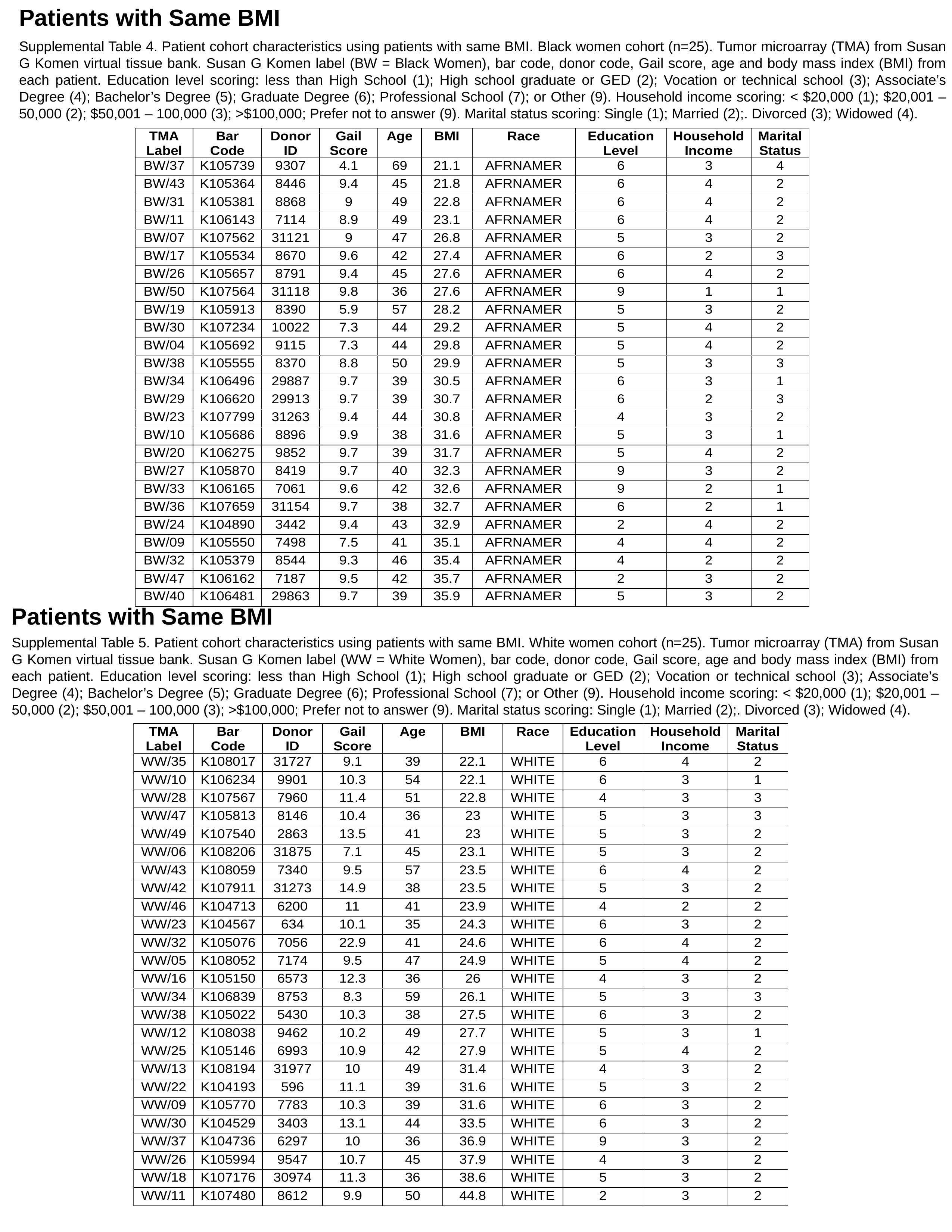

Patients with Same BMI
Supplemental Table 4. Patient cohort characteristics using patients with same BMI. Black women cohort (n=25). Tumor microarray (TMA) from Susan G Komen virtual tissue bank. Susan G Komen label (BW = Black Women), bar code, donor code, Gail score, age and body mass index (BMI) from each patient. Education level scoring: less than High School (1); High school graduate or GED (2); Vocation or technical school (3); Associate’s Degree (4); Bachelor’s Degree (5); Graduate Degree (6); Professional School (7); or Other (9). Household income scoring: < $20,000 (1); $20,001 – 50,000 (2); $50,001 – 100,000 (3); >$100,000; Prefer not to answer (9). Marital status scoring: Single (1); Married (2);. Divorced (3); Widowed (4).
Patients with Same BMI
Supplemental Table 5. Patient cohort characteristics using patients with same BMI. White women cohort (n=25). Tumor microarray (TMA) from Susan G Komen virtual tissue bank. Susan G Komen label (WW = White Women), bar code, donor code, Gail score, age and body mass index (BMI) from each patient. Education level scoring: less than High School (1); High school graduate or GED (2); Vocation or technical school (3); Associate’s Degree (4); Bachelor’s Degree (5); Graduate Degree (6); Professional School (7); or Other (9). Household income scoring: < $20,000 (1); $20,001 – 50,000 (2); $50,001 – 100,000 (3); >$100,000; Prefer not to answer (9). Marital status scoring: Single (1); Married (2);. Divorced (3); Widowed (4).

## Slide 20
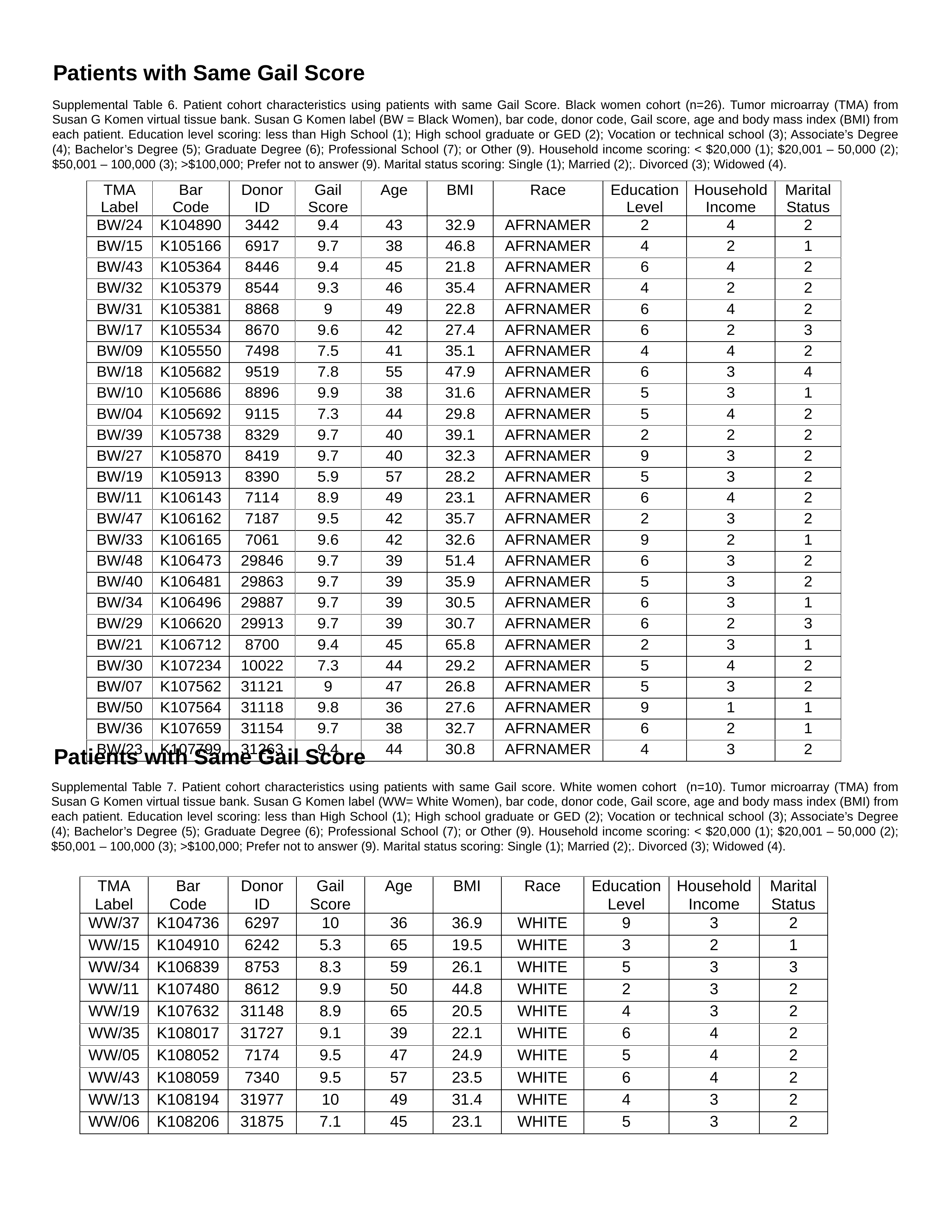

Patients with Same Gail Score
Supplemental Table 6. Patient cohort characteristics using patients with same Gail Score. Black women cohort (n=26). Tumor microarray (TMA) from Susan G Komen virtual tissue bank. Susan G Komen label (BW = Black Women), bar code, donor code, Gail score, age and body mass index (BMI) from each patient. Education level scoring: less than High School (1); High school graduate or GED (2); Vocation or technical school (3); Associate’s Degree (4); Bachelor’s Degree (5); Graduate Degree (6); Professional School (7); or Other (9). Household income scoring: < $20,000 (1); $20,001 – 50,000 (2); $50,001 – 100,000 (3); >$100,000; Prefer not to answer (9). Marital status scoring: Single (1); Married (2);. Divorced (3); Widowed (4).
Patients with Same Gail Score
Supplemental Table 7. Patient cohort characteristics using patients with same Gail score. White women cohort (n=10). Tumor microarray (TMA) from Susan G Komen virtual tissue bank. Susan G Komen label (WW= White Women), bar code, donor code, Gail score, age and body mass index (BMI) from each patient. Education level scoring: less than High School (1); High school graduate or GED (2); Vocation or technical school (3); Associate’s Degree (4); Bachelor’s Degree (5); Graduate Degree (6); Professional School (7); or Other (9). Household income scoring: < $20,000 (1); $20,001 – 50,000 (2); $50,001 – 100,000 (3); >$100,000; Prefer not to answer (9). Marital status scoring: Single (1); Married (2);. Divorced (3); Widowed (4).
